# Prediction Pipeline Selection for Incomplete Clinical Data via Missingness Fingerprints and Instance Augmentation

**DOI:** 10.3390/bioengineering13050497

**Published:** 2026-04-24

**Authors:** Runze Li, Zhuyi Shen, Chengkai Wu, Jingsong Li, Yu Tian

**Affiliations:** 1 College of Biomedical Engineering & Instrument Science, Zhejiang University, Hangzhou 310027, China; lrzlr2020@zju.edu.cn; 2 School of Intelligent Science and Technology, Hangzhou Institute for Advanced Study, University of Chinese Academy of Sciences, Hangzhou 310024, China; shenzhuyi@ucas.ac.cn (Z.S.); wuck@ucas.ac.cn (C.W.)

**Keywords:** algorithm selection, electronic health records, missing data, graph neural networks, meta-learning, instance space analysis, clinical prediction

## Abstract

Clinical prediction from electronic health records (EHRs) is complicated by pervasive missingness and label scarcity, which make performance sensitive to the match between data conditions and pipeline choice. Choosing the best pipeline for a new incomplete dataset still requires costly trial-and-error. We cast this as an algorithm selection problem and address two bottlenecks—instance scarcity and distance quality—that have so far prevented meta-learning from reaching clinical settings. Graph neural networks offer diverse strategies (patient similarity networks, bipartite imputation graphs, attention-driven feature interaction), yet no single architecture dominates across missingness patterns, and selecting the best pipeline for a new dataset remains a trial-and-error approach. Formal algorithm selection could automate this choice but requires many characterized meta-instances—more than clinical settings typically provide. We propose two solutions: (1) constructive instance augmentation, applying controlled quality perturbations (MCAR and MNAR missingness injection, label trimming) to 20 base EHR datasets to expand the meta-knowledge base to 83 characterized meta-instances, each described by a 10-dimensional missingness fingerprint, without additional model training; and (2) dynamic-supervised metric learning, using differential evolution to optimize fingerprint feature weights so that static distances preserve method-performance similarity captured by dynamic fingerprints, which require model sweeps and are unavailable at deployment. Under base-dataset-level leave-one-dataset-out cross-validation over 21 pipelines, the resulting metric-learned kNN recommender attains the highest win rate (20.5%) among non-oracle strategies on the augmented store, selecting the correct pipeline more often than any fixed default. At deployment, the recommender needs only the 10-dimensional static fingerprint with pre-learned weights; no sweep data is required for new datasets. Cross-domain evaluation on 25 external subsets (colorectal cancer, kidney disease, MIMIC-IV) demonstrates framework modularity: when the fingerprint module is adapted (standard meta-features in place of the missingness-specific set), the recommender achieves regret of 0.025 (55% below random selection).

## 1. Introduction

Electronic health records (EHRs) are pervasively incomplete. Lab values go unordered, vitals are intermittently recorded, and diagnostic labels are scarce. The resulting missingness patterns vary across institutions, ICU types, and patient populations [1,2]. Clinical prediction models are highly sensitive to this variation: a pipeline that excels on one missingness profile may fail on another.

Graph neural networks (GNNs) have been applied to clinical tabular data by modeling patient–patient similarity [3], bipartite feature–observation structure [4], or attention-driven variable interaction [5]. The field is fragmented: architectures such as GIN [6], GCNII [7], and H2GCN [8] have complementary inductive biases, and combined pipelines chain modules in sequence. No single architecture dominates across missing rates, mechanisms, and label availability—our benchmarking results are consistent with this general intuition, as formalized by the No Free Lunch theorems [9] under idealized assumptions (uniform prior over all possible problems) that are stronger than what clinical data presents.

These methods span three complementary strategies—patient similarity graphs, bipartite imputation graphs, and attention-driven architectures (Section 2.2)—each with different strengths under varying missingness and label conditions. Their non-linear modeling of inter-patient and inter-feature relationships can outperform conventional impute-then-classify pipelines, particularly when missingness patterns carry clinical signal.

At the same time, AI-based analysis of clinical signals—ECG interpretation [10], EEG-based neurological assessment [11], and AI-assisted management of chronic conditions such as hypertension [12]—is generating new derived features that are increasingly stored in EHR systems. As these features accumulate, missing-data patterns will grow more complex, which further motivates pipeline selection methods that can adapt to heterogeneous data-quality profiles.

Choosing which pipeline to deploy for a new dataset is therefore still trial-and-error. Rice [13] formalized this as the Algorithm Selection Problem (ASP): map problem instances, characterized by measurable features, to the algorithm most likely to maximize performance. Portfolio-based selectors such as SATzilla [14] and the ASlib benchmark library [15,16] produced large gains in combinatorial optimization, where performance spreads span orders of magnitude. Formal algorithm selection has not, however, been applied to clinical machine learning. A recent survey [17] found only seven prior works since 2005 on automated imputation method selection, none using clinical data.

Where prior AutoML work concentrates on per-dataset hyperparameter tuning [18], our framework operates one level higher: it recommends *which* pipeline to deploy, not how to tune it. The two are complementary—a shortlist of 3–5 pipelines from the recommender can seed a targeted AutoML search, reducing the candidate space from 21 methods. We address this gap with the first missingness-aware meta-feature set and the first instance augmentation protocol tailored to EHR data quality.

The obstacle is practical: meta-learning requires a library of characterized datasets with known method performance, and clinical groups typically have only a handful. The StatLog project used 22 datasets, Brazdil et al. [19] used 53, and the pymfe toolkit [20] includes no features for missing data. With so few instances and no missingness-aware features, learned selectors cannot outperform a robust default.

**Figure 1:**
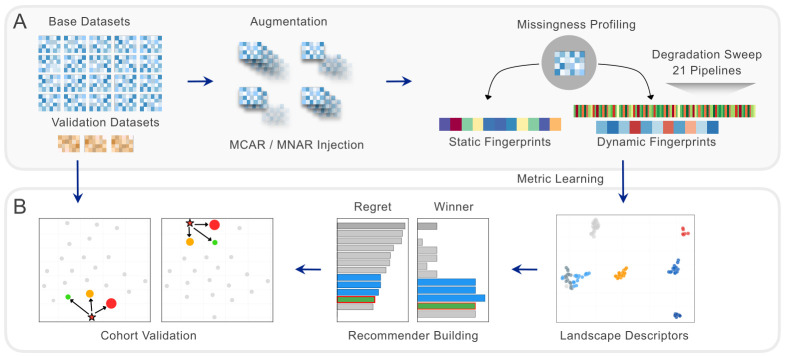
Overview of the proposed framework. (**A**) Benchmarking infrastructure: 20 EHR datasets are evaluated with 21 methods under a controlled degradation sweep; winner maps (right) show the best method per condition. (**B**) Meta-learning framework and deployment: static fingerprints and instance augmentation build the knowledge base (left); metric learning optimizes feature weights using sweep-derived performance vectors as supervision (center); at deployment, weighted *k*-NN returns a method recommendation from the knowledge base (right).

We address both gaps—instance scarcity and distance quality—through constructive instance augmentation and dynamic-supervised metric learning. Controlled quality perturbations (MCAR and MNAR missingness injection, label trimming) transform 20 base EHR datasets into 83 characterized meta-instances, each described by a 10-dimensional missingness fingerprint (with Little’s MCAR test statistic [21] as an 11th dimension for stored instances). The idea draws on perturbation-response profiling in single-cell biology, where molecular perturbations expose functional states that surface markers miss [22]. Here, quality perturbations expose data-quality structures that a global missing rate alone does not: each perturbed version occupies a distinct region of the fingerprint space, with a different optimal method and performance profile. The resulting dynamic fingerprints—per-method performance vectors that require the full sweep—then supervise metric learning on the static fingerprint, optimizing feature weights so that static distances preserve method-performance similarity. At deployment, only the ten-dimensional static fingerprint with pre-learned weights is needed. Figure 1 gives an overview of the proposed framework.

The contributions are: (1) a controlled benchmarking framework evaluating 21 pipelines across missingness × mechanism × label rate; (2) a 10-dimensional missingness fingerprint (with Little’s MCAR test as a stored 11th dimension that does not enter the distance computation), the first meta-feature set designed for clinical missing data [17,20]; (3) constructive instance augmentation expanding the meta-knowledge base from 20 to 83 characterized instances, plus 10 synthetic datasets with explicit MAR-mechanism coverage (30 base datasets total; Section 4.1); (4) cross-domain validation on 25 external subsets (colorectal cancer, kidney disease, MIMIC-IV) demonstrating that the fingerprint module is pluggable—substituting pymfe meta-features reduces cross-domain regret to 0.025, 55% below random (Section 4.8); and (5) dynamic-supervised metric learning that transfers sweep-derived information into static fingerprint weights so that deployment requires no model runs on new datasets. All five are evaluated with anti-leakage base-dataset-level leave-one-dataset-out (LODO) cross-validation.

Section 2 reviews related work. Section 3 and Section 4 describe methods and results. Section 5 discusses the findings and limitations, and Section 6 concludes this study.

## 2. Related Work

### 2.1. Algorithm Selection and Meta-Learning

In Rice’s ASP framework [13], measurable instance features guide the choice of algorithm. The No Free Lunch theorems [9] motivate this: no single algorithm dominates universally, so instance-aware selection is necessary in principle. Whether it helps in practice depends on the VBS-SBS gap, the performance difference between the Virtual Best Solver (oracle per-instance selection) and the Single Best Solver (best fixed algorithm). Tornede et al. [23] showed on the ASlib benchmark library [15] that meta-learning yields gains only when this gap is wide enough; narrow gaps defeat learned selectors.

The ASP framework has a long history in combinatorial optimization and machine learning. SATzilla [14] showed that portfolio-based selection can dominate individual SAT solvers when performance spreads span orders of magnitude. ASlib [15] standardized the evaluation protocol for cross-domain comparison [16], and Kerschke et al. [24] surveyed the resulting methods. Brazdil et al. [19] established instance-based (*k*-nearest-neighbor, kNN) meta-learning as effective with small meta-datasets, using dataset-level features to predict algorithm rankings. On the feature side, Pfahringer et al. [25] introduced landmarking—using the performance of cheap probe algorithms as meta-features—and drew the distinction between static features (computed from data alone) and dynamic features (requiring model runs).

Smith-Miles and Munoz [26] developed Instance Space Analysis (ISA), a methodology for visualizing algorithm performance footprints across problem instances. ISA has been applied to over 30 domains but never to healthcare prediction. Auto-sklearn [27] uses meta-learning to warmstart AutoML, while AutoPrognosis [18] performs per-dataset search rather than building reusable instance-to-algorithm mappings. Soares [28] proposed datasetoids—synthetic meta-instances generated by permuting target and feature roles within a dataset. This increases instance count but does not alter the data-quality profile: all datasetoids inherit the same missing rate, mechanism, and label availability as their parent. Our constructive augmentation deliberately modifies these quality dimensions, producing instances that may have different optimal methods and that occupy distinct regions of the fingerprint space. The distinction matters because algorithm selection for missing data requires meta-instances that vary in data-quality conditions, not merely in feature semantics.

Few-shot learning offers a parallel: task augmentation—synthesizing novel tasks from existing ones—expands the meta-training distribution and improves generalization to unseen tasks [29,30]. Our approach augments the instance space by modifying data-quality conditions rather than the task space, producing meta-instances with different optimal pipelines rather than different classification episodes.

### 2.2. Graph Methods for Clinical Prediction

Graph neural networks have been applied to clinical tabular data by modeling relationships between patients, features, or both. Patient similarity networks (PSNs) [3] construct graphs where nodes are patients and edges encode feature-space proximity, enabling label propagation and semi-supervised learning when labeled samples are scarce. GRAPE [4] takes a bipartite approach, treating patients and features as separate node sets with observed values as edge attributes, and handles missingness through message passing. The Graph Convolutional Transformer (GCT) [5] uses attention-based feature interaction to jointly learn graphical structure among clinical variables and perform supervised prediction.

General-purpose GNN architectures bring different inductive biases to patient-similarity graphs. GIN [6] maximizes expressive power among message-passing networks. GCNII [7] uses initial residual connections and identity mapping for deep architectures without oversmoothing. APPNP [31] decouples prediction from propagation via personalized PageRank. H2GCN [8] targets heterophilous graphs where connected nodes may have different labels, a plausible scenario in patient similarity networks where clinically dissimilar patients share surface-level features. Recent graph transformers (GraphGPS [32], Exphormer [33], NAGphormer [34]) target much larger graphs than our patient-similarity scale (typically N<2000). No existing benchmark, however, systematically evaluates these architectures under controlled quality degradation—varying missing rates, mechanisms, and label availability.

Section 3.3 describes how these architectures enter our pipeline portfolio in three integration patterns—graph-as-imputer, graph-as-encoder, and hybrid—with the recommender selecting among them based on missingness profile.

### 2.3. Missing Data in Clinical Records

Rubin [35] defined the standard taxonomy of three missingness mechanisms: Missing Completely At Random (MCAR), Missing At Random (MAR), and Missing Not At Random (MNAR). In clinical data, the mechanism is rarely known and often mixed: lab values may be missing because they were not ordered (MAR, conditional on clinical suspicion) or because the result was normal (MNAR, conditional on the unobserved value itself). Li et al. [36] found that integrating genetic information with clinical variables improves EHR imputation accuracy, indicating that missingness in clinical records is structured rather than purely random and that auxiliary data sources can exploit this structure.

A recent perspective [2] frames EHR incompleteness as a systemic process driven by patient, provider, technology, and policy factors, and proposes the Record Strength Score as a composite completeness metric. Yet, a systematic review of 46 EHR imputation studies found that MCAR—the simplest and least realistic mechanism—was the most commonly simulated (63%) [1]. Jager et al. [37] benchmarked imputation methods across MCAR, MAR, and MNAR but did not explore mechanism-aware method recommendation. Little’s MCAR test [21] assesses whether the observed missingness pattern is consistent with MCAR, providing a data-driven mechanism label without ground truth. The test has known limitations: low power when unique missingness patterns are numerous, and the inability to distinguish MAR from MNAR. We use Little’s test as one dimension ($f_{11}$) of the missingness fingerprint, alongside structural dimensions ($f_{1}$–$f_{10}$) that no existing meta-feature toolkit provides [17,20].

## 3. Materials and Methods

### 3.1. Problem Formulation

We instantiate Rice’s Algorithm Selection Problem (ASP) framework [13] for EHR prediction with four components. (i) Problem space P: clinical datasets, both natural and quality-perturbed (Section 3.6); each is a binary classification task (e.g., in-hospital mortality). (ii) Feature space F: a 10-dimensional missingness fingerprint (Section 3.4) computed from the data alone, with Little’s MCAR test as an 11th stored dimension that does not enter the kNN distance computation. (iii) Algorithm space A: 21 candidate pipelines—imputation-based, graph-based, and end-to-end (Section 3.3). (iv) Performance mapping: AUROC averaged over 30 stratified 80/20 train/test splits, preserving class balance. Unlabeled samples (after label trimming) enter the training set for semi-supervised methods but are excluded from evaluation; no separate validation set is held out and all hyperparameters use fixed defaults. Given only a dataset’s fingerprint, the recommender predicts which pipeline will yield the highest AUROC.

### 3.2. Datasets

Table 1 summarizes the 30 datasets used in this study.

The 20 base subsets come from eICU-CRD v2.0 [38], a multi-center critical-care database covering over 200 US hospitals. Each subset is defined by ICU unit type and primary diagnosis, with 27 clinical features (vitals, labs, comorbidities, Glasgow Coma Scale [GCS]). The subsets share a common feature schema but differ in missingness structure (native missing rates 4–13%): lab-ordering practices, documentation conventions, and patient acuity vary across ICU types and contributing institutions so that subsets from surgical ICUs, cardiac care units, and neurological ICUs occupy distinct regions of the fingerprint space despite the shared features. External validation relies on two independent cohorts that were not used during knowledge base construction: FAHZU-Kidney (N=14,164, 28 features, 43.7% missing; hemodialysis patients, Zhejiang, China) and SAHZU-CRC (N=2080, 18 features, 5.5% missing; colorectal cancer, Zhejiang, China), plus 8 MIMIC-IV ICD-diagnosis groups (N=1771–24,873, 28 features, 15–29% missing; US multi-center ICU [39]). Together, these span different variable schemas (18–28 features), institutions, countries, and clinical specialties, providing out-of-distribution tests for the fingerprint-based recommender.

### 3.3. Candidate Methods

Twenty-one pipelines in two paradigm groups are shown in Table 2.

PSN-SGAN (Patient Similarity Network with Semi-supervised GAN) [47] constructs a patient similarity graph via *k*-nearest neighbors (k=30, Euclidean distance) with Jaccard edge refinement, and then trains a semi-supervised GAN on graph-augmented representations. The generator is a three-layer MLP ($z\;=\;200\to 500\to 500\to F+d_{\mathrm{emb}}$) with batch normalization; the discriminator is a five-layer weight-normalized MLP (500-500-250-250-250) trained with Wasserstein loss and gradient penalty ($\lambda_{\mathrm{gp}}\;=\;10$). Node2Vec embeddings (64-dim) are concatenated with raw features as the discriminator input.

Combined-A and Combined-B chain the GCT encoder [5] and PSN-SGAN in opposite orders. The GCT encoder uses per-feature linear projections with learnable mask embeddings for missing values, two transformer layers with correlation-guided attention, and a [CLS] token whose final hidden state serves as a 64-dimensional patient embedding. Combined-A (GCT→PSN-SGAN) first extracts GCT [CLS] embeddings from the raw features, and then builds the patient similarity graph and runs PSN-SGAN classification in the embedding space. Combined-B (PSN-SGAN→GCT) first trains PSN-SGAN to generate pseudo-labels for unlabeled samples (confidence threshold 0.8), and then trains GCT with the augmented label set. Full architectural and training details are provided in Supplementary Section S1; hyperparameter settings for all 21 pipelines are listed in Section S2. All hyperparameters for Combined-A and Combined-B are inherited directly from their constituent modules: the GCT encoder uses 2 transformer layers, 64-dim embeddings, and learning rate $10^{-3}$ with cosine annealing; PSN-SGAN uses k=30, Wasserstein loss, gradient penalty λ=10, and discriminator-to-generator step ratio $n_{\mathrm{critic}}\;=\;5$. No hyperparameter was tuned on the eICU evaluation datasets; the same fixed settings are applied across all 20 subsets and all degradation conditions. The confidence threshold for pseudo-label generation in Combined-B (0.8) is the original PSN-SGAN default. Training uses Adam (GCT: $\mathrm{lr}\;=\;10^{-3}$, cosine annealing, gradient clipping ${∥\cdot ∥}_{\max}\;=\;1.0$; PSN-SGAN: $\mathrm{lr}\;=\;3\times 10^{-3}$, $\beta_{1}\;=\;0.5$) with early stopping (patience 20 epochs) for both modules. GCT minimizes binary cross-entropy plus an attention-regularization term (KL divergence between consecutive-layer attention maps, weight 0.1); PSN-SGAN trains the discriminator $n_{\mathrm{critic}}\;=\;5$ steps per generator step, using exponential moving average (decay 0.999) for parameter updates. In Combined-B, pseudo-labels are generated by applying the PSN-SGAN discriminator’s class posterior to unlabeled samples, retaining those with $\max(\hat{p})\geq 0.8$; GCT then trains on the union of original and pseudo-labeled samples.

The Combined-A (GCT→PSN-SGAN) ordering is better suited to informative missingness. Because GCT uses learnable mask embeddings, its attention layers treat missing and observed values differently, and the [CLS] embeddings that result encode both feature magnitudes and patterns of absence. PSN-SGAN then builds the patient similarity graph over these embeddings; so, *k*NN neighbours share similar missingness patterns as well as similar observed values. In Combined-B (PSN-SGAN→GCT), the patient graph is built on raw features, where Euclidean distance does not distinguish a missing value from a low one. The pseudo-labels from this graph carry less information about missingness, and GCT receives the augmented label set without the graph’s structural signal. Combined-A should therefore dominate when missingness is outcome-informative (i.e., under MNAR). Section 4.1 quantifies this effect across all 84 conditions.

End-to-end methods handle missing values internally: XGB-Native exploits sparsity-aware split finding, and FT-Transformer uses per-feature tokenization with learned mask embeddings.

### 3.4. Missingness Fingerprint

Ten dimensions enter the kNN distance computation; an eleventh (Little’s MCAR test, $f_{11}$) is stored for analysis but excluded from the recommender (Table 3).

No existing meta-feature toolkit provides missing-data-specific features [17,20]. Dimensions $f_{1}$–$f_{10}$ are computed from the data matrix alone in under one second. $f_{11}$ (Little’s MCAR test) is appended during instance augmentation (Section 3.6), completing the 11-dimensional representation for the augmented knowledge base. The recommender uses $f_{1}$–$f_{10}$ with learned weights (Section 3.9) for kNN lookup. $f_{11}$ characterizes the mechanism signature of each stored instance but does not enter the distance computation: the ten structural dimensions describe the static missingness profile, while $f_{11}$ is a statistical mechanism indicator.

$f_{3}$ normalizes by 0.25, the maximum variance in a Bernoulli variable (attained at p=0.5), so that $f_{3}\in \left[0,1\right]$ where 1 indicates maximally heterogeneous per-feature missing rates. $f_{9}$ can approach 1 for small datasets (e.g., N=76 for eicu-18) where most patients have unique patterns. In practice, this reflects genuine structural diversity in small cohorts rather than noise, but it means $f_{9}$ is more informative for relative comparisons across datasets of similar size.

Dimension selection rationale: The 10 structural dimensions cover three axes of the missingness profile: amount ($f_{1}$, $f_{3}$, $f_{4}$: global rate, cross-feature variance, and per-feature maximum), structure ($f_{5}$, $f_{6}$, $f_{7}$: inter-feature and feature-label missingness correlations), and diversity ($f_{9}$, $f_{10}$: unique pattern count and concentration), with $f_{2}$ (label rate) and $f_{8}$ (class imbalance) as task-level descriptors that modulate method behavior independently of missingness. We deliberately exclude sample size *N* and feature count *F*. In the augmentation framework, all perturbed versions of a base dataset share the same *N* and *F*; so, these dimensions add no discriminative power for distinguishing quality conditions within a dataset while potentially causing the recommender to match by cohort size rather than by missingness structure. Similarly, distributional features (skewness, outlier proportion) describe value distributions rather than missingness patterns; while they may influence imputation accuracy, they conflate feature semantics with data-quality characterization, which the fingerprint deliberately avoids. Whether adding *N*, *F*, or distributional features further reduces regret is an empirical question; we report this as a planned ablation in Section 5.5.

Framework modularity: The recommender framework is modular: the fingerprint is a pluggable component. Ablation experiments (Supplementary Table S4) demonstrate that standard meta-features (pymfe [20], 6 dimensions: class_conc.sd, freq_class.sd, class_ent, class_conc.mean, mad.sd, attr_conc.sd; a superset pymfe8 adds joint_ent.mean and min.sd) achieve lower cross-domain regret (0.025 vs. 0.066 for $f_{1}$–$f_{10}$) when paired with a synthetic-only knowledge base, because four of the ten missingness-specific dimensions ($f_{2}$, $f_{7}$, $f_{8}$, $f_{9}$) exhibit poor overlap between eICU and external datasets. The $f_{1}$–$f_{10}$ fingerprint provides interpretable, domain-specific characterization and competitive in-distribution performance (regret 0.039 vs. random 0.061). The framework’s value lies in the augmentation-and-recommendation architecture, not in a specific feature set; practitioners can substitute any feature extractor suited to their deployment setting.

### 3.5. Degradation Sweep

Controlled perturbations: missingness injection 0–60% under MCAR/MNAR, label trimming 5–100%.

- MCAR injection: Bernoulli dropout, p=(t−c)/(1−c), where *t* is the target rate and *c* is the current rate [50]. This mimics sensor failures, random transcription errors, and other non-systematic omissions in clinical documentation; the resulting missingness is independent of both observed and unobserved values.
- MNAR injection: Noisy rank-threshold masking—bottom quantile of each column’s observed values—is masked with Gaussian rank noise (σ=0.15) for stochasticity [50,51]. The left-tail masking approach is motivated by clinical MNAR patterns where low values are selectively unrecorded. For instance, albumin may not be ordered for patients presumed well-nourished, or hemoglobin tests may be skipped when anemia is not suspected. Because the missingness depends on the unobserved value itself, the resulting absence patterns are informative and can bias downstream prediction.
- MAR is not separately injected. MAR and MNAR cannot be distinguished from observed data alone [35]; no test, including Little’s [21], can separate them without access to unobserved values. Clinical EHR missingness is typically a MAR/MNAR mixture (e.g., lab ordering conditional on clinical suspicion, or values missing because they are normal). Rather than simulating a mechanism that cannot be verified, our augmentation preserves the native mixture in each base dataset. The injection rates in Table 4 are calibrated to avoid overwhelming the original structure: baseline and light-injection instances retain their native non-MCAR signature (Section 4.4, 74–79% rejection rate), confirming that the pre-existing missingness structure, including any non-MCAR component, is not overwritten.
- MAR coverage via synthetic instances: Beyond MCAR and MNAR injection, the knowledge base contains synthetic instances whose missingness is MAR by construction. A conditional probability chain generates them: a patient’s diagnosis determines which procedures are ordered (diagnosis→procedure probabilities), and the ordered procedures determine which lab results are observed (procedure→lab probabilities). A lab value is absent because the corresponding procedure was never ordered for that diagnosis—missingness depends on an observed variable (the diagnosis), not on the missing value itself, which is the defining property of MAR. In the fingerprint space, these synthetic instances occupy regions distinct from both MCAR-injected and MNAR-injected instances. Together with the native MAR component already present in eICU data (74–79% MCAR rejection rate; Section 4.4), the knowledge base now covers all three standard mechanisms: MCAR (injection), MAR (synthetic + native), and MNAR (left-tail injection).
- Sweep grid: 7 missing rates × 6 label rates × 2 mechanisms × 21 methods × up to 30 repeats per dataset, totaling approximately 1,060,000 individual model evaluations across all datasets.

**Figure 2:**
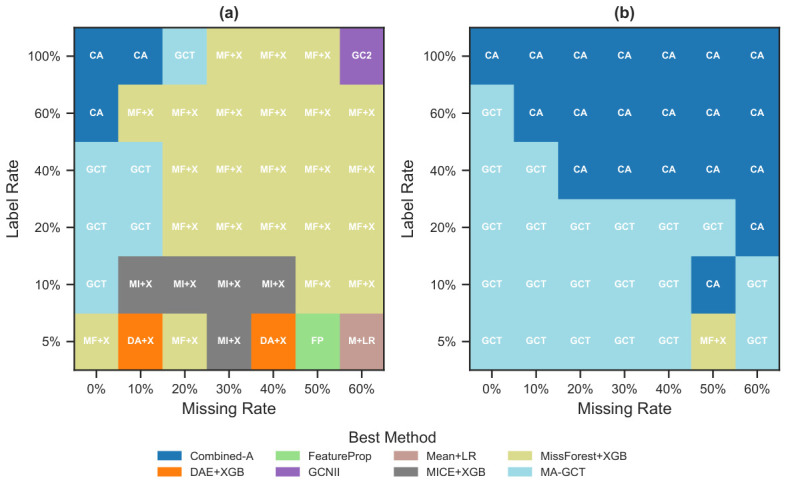
Winner maps showing the best method in each missing rate × label rate cell, averaged across 20 datasets. Cells are colored by the winning method and annotated with its abbreviation. (**a**): MCAR; (**b**): MNAR. Panel labels indicate mechanism type.

The resulting winner maps are shown in Figure 2.

### 3.6. Constructive Instance Augmentation

For each base dataset with sweep data, we select six conditions (Table 4) spanning the quality space. For each: (i) physically degrade the data; (ii) compute the 10-dimensional static fingerprint and append $f_{11}$ (Little’s MCAR test); and (iii) look up method AUCs from the existing sweep checkpoint. Not all datasets have sweep data at every condition: heavy conditions (miss ≥ 0.5) require grid points that only 6 of the 20 base datasets currently cover; so, the actual number of augmented instances varies by condition (see Section 4.6). From 120 candidate augmentations, 37 are excluded for insufficient sweep coverage or degenerate method performance at extreme grid points, leaving 83 meta-instances produced in under 5 min with no additional model training.

**Figure 3:**
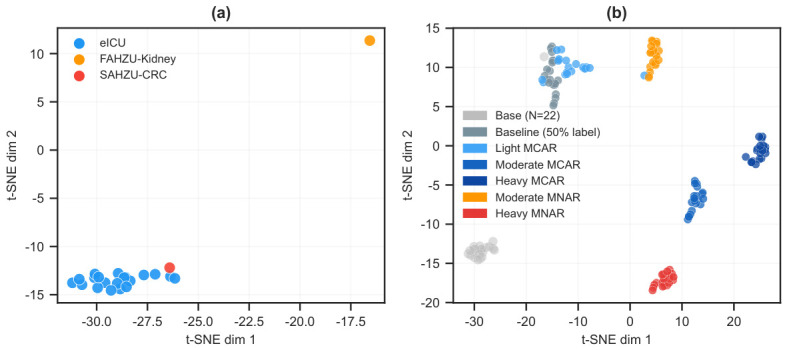
Instance space visualization via t-SNE on the 11-dimensional static fingerprint. (**a**) Base and external datasets (N=22); external cohorts (FAHZU-Kidney, SAHZU-CRC) are labeled. (**b**) After augmentation (N=83), instances are colored by degradation condition. The augmented store covers a wider region of the fingerprint space than the base datasets alone.

The six conditions in Table 4 span two orthogonal quality axes—missing rate and label availability—under both MCAR and MNAR. Injection ranges from 0% (baseline) to 50% (heavy MCAR); MNAR conditions use 20% and 40%. Label rates are paired inversely with missing rates (e.g., heavy MCAR combines 50% missingness with only 10% labels) so that the resulting instances populate distinct fingerprint regions without reaching degenerate settings where all methods collapse. Figure 3b confirms the intended separation: MCAR-injected instances spread along $f_{1}$ (global missing rate), while MNAR-injected instances show elevated $f_{5}$ (missingness correlation). Of the 120 candidate augmentations (6×20 datasets), 37 were dropped for insufficient sweep coverage or degenerate performance, leaving 83 viable meta-instances.

Each perturbed version has a distinct fingerprint. A 30% MNAR injection elevates $f_{5}$ (missingness correlation) and changes $f_{11}$ (Little’s test), placing the instance in a different fingerprint region than its MCAR counterpart. We refer to the 20 base eICU datasets (18 with complete sweep coverage at the reference condition mr=0.2,lr=0.4, MCAR) as the original store and the 83 augmented instances as the augmented store.

The augmented store (N=83) derives from the 20 eICU base datasets under six perturbation conditions (Table 4); 37 of the 120 candidate augmentations are excluded for insufficient sweep coverage, leaving instances from 19 base groups. The 10 synthetic datasets (Section 3.5) are not augmented; they are used only as an alternative knowledge base for cross-domain evaluation (Section 4.8). Combined with eICU, this gives 30 base datasets in total.

### 3.7. Anti-Leakage Evaluation

Perturbed versions of the same base dataset share underlying structure. We use base-dataset-level LODO: when testing any instance from base *B*, all instances from *B* are excluded. This evaluates generalization to unseen clinical populations. Under this protocol we evaluate eight recommender strategies (Table 5), ranging from random and always-one-method baselines to the proposed Dynamic-Learned-kNN.

### 3.8. Landscape Descriptors

We first tried a per-method dynamic fingerprint (31 dimensions: one degradation slope per method per mechanism), but kNN performed below random selection—31 dimensions are too many for ∼80 meta-instances. The full method×condition performance matrix (∼1600 dimensions) fared worse still. Instead, we extract ten method-agnostic landscape descriptors that capture the shape of the performance surface without reference to specific methods.

Ten method-agnostic dimensions from the sweep surface: baseline/stress composite dispersion, mean AUROC/Brier degradation slopes, MNAR-MCAR gap, winner stability and diversity, label efficiency spread, Pareto frontier size, and calibration discordance. These cannot be computed for unseen datasets; their role is analytical via decoupling analysis. In addition, the per-method performance vectors from the sweep define dynamic distances between instances, which serve as the supervision signal for metric learning (Section 3.9).

### 3.9. Dynamic-Supervised Metric Learning

The landscape descriptors and per-method performance vectors from the degradation sweep encode how methods respond to each dataset’s conditions. These dynamic fingerprints capture a method-performance structure that static fingerprints alone miss, but they require the full sweep and are unavailable for new datasets. We use them as a supervision signal to learn feature weights for the static fingerprint, transferring dynamic-level discrimination into a representation computable without model runs.

Concretely, let $s_{i}\in R^{10}$ be the static fingerprint of instance *i* and $d_{ij}^{\mathrm{dyn}}$ the Euclidean distance between dynamic performance vectors of instances *i* and *j*. We seek non-negative weights $w\in R_{\geq 0}^{10}$ ($\sum w_{k}=1$) that maximize Spearman’s rank correlation between the weighted static distances $d_{ij}^{\mathrm{static}}\left(w\right)=∥w⊙\left(s_{i}-s_{j}\right)∥$ and $d_{ij}^{\mathrm{dyn}}$, with a secondary nearest-neighbour method-match reward. The combined objective is $-(0.7\;\rho +0.3\;r_{\mathrm{match}})$, where ρ is Spearman’s correlation and $r_{\mathrm{match}}$ the fraction of nearest-neighbour method matches. Optimization uses scipy.optimize.differential_evolution with strategy best1bin, population size 15, mutation factor (0.5,1.0), crossover rate 0.7, 500 iterations, tolerance $10^{-8}$, and fixed seed 42 [52]. Weights are parameterized in log-space (bounds ${\left[-3,3\right]}^{10}$) and simplex-normalized after optimization.

To avoid data leakage, weights are re-learned in each LODO fold, excluding all instances from the held-out base dataset. Across folds, the learned weights consistently concentrate on $f_{1}$ (global missing rate, median 47%) and $f_{9}$ (pattern diversity, median 37%), with remaining dimensions receiving <5% each. This is interpretable: missing rate and pattern diversity are the two structural properties most predictive of which imputation strategy will succeed, consistent with the mechanism dichotomy identified in Section 4.7.

Implications of weight concentration: The concentration on two dimensions does not imply the remaining eight are dispensable. First, the weights are medians across LODO folds; individual folds assign up to 12% to secondary dimensions (e.g., $f_{5}$ in folds where inter-feature missingness correlation distinguishes methods). Second, the condition-matching ablation (Section 5.1) shows that masking $f_{1}$, $f_{2}$, and $f_{11}$ increases regret by only 0.001, meaning the structural dimensions $f_{3}$–$f_{10}$ carry sufficient signal to sustain near-full performance when the dominant dimensions are removed. The 10-dimensional representation thus provides redundancy that guards against edge cases where $f_{1}$ and $f_{9}$ alone would be insufficient—for instance, datasets with similar global missing rate and pattern diversity but different inter-feature missingness correlations ($f_{5}$, $f_{6}$).

At deployment, the recommender requires only the 10-dimensional static fingerprint (<1 s to compute) with the learned weights applied before kNN lookup, with no sweep, no dynamic fingerprint, or additional model training.

## 4. Results

### 4.1. Method Complementarity

Before the per-method analysis, we note that our injection protocol produces the intended mechanism labels: Little’s MCAR test rejects MCAR for baseline and light-injection instances but not under heavier injection (Table 6, full discussion in Section 4.4).

The winner maps (Figure 2) confirm that no single method dominates across all conditions. Across the full 84-cell condition space (7 miss × 6 label × 2 mechanisms), MA-GCT wins 27 cells (32.1%), followed by MissForest+XGB with 24 (28.6%) and Combined-A with 23 (27.4%). The remaining wins are distributed among MICE+XGB (5, 6.0%), DAE+XGB (2, 2.4%), FeatureProp, Mean+LR, and GCNII (1 each, 1.2%). Thirteen of 21 methods never win any cell. The top three winners split the condition space roughly by mechanism: missingness-aware methods (MA-GCT + Combined-A) dominate MNAR, MissForest+XGB dominates MCAR, and no single method exceeds 55% of wins under either mechanism—confirming strong complementarity.

The missingness mechanism is the strongest predictor of winner identity: under MCAR (42 cells), winners are dispersed across eight methods (H=2.11 bits), whereas under MNAR, only three methods win (H=1.14 bits), with missingness-aware architectures (MA-GCT + Combined-A) jointly accounting for 41 of 42 cells. Section 4.7 provides the full regime-stratified analysis. The caveat that this dichotomy reflects our specific injection protocol is also discussed there. (Grid-level winners are determined after averaging AUROCs across datasets; so, a method that never tops any averaged cell can still be the best on individual datasets.) When restricted to the LODO evaluation conditions (Section 3.7), 11 of 21 methods win on at least one dataset, with MissForest+XGB (5 wins), Mean+XGB (3), and five others (2 each) leading. The lack of a clear single best solver under evaluation conditions supports instance-adaptive selection.

Across the full per-dataset grid (84 conditions × 30 KB datasets), all 21 methods win at least one cell (Supplementary Figure S7); the competitive ranking is condition-dependent throughout. Ten methods that never win at the specific LODO evaluation condition (miss =0.2, label =0.4, MCAR) nonetheless reach the top 3 in other conditions, which argues for condition-aware selection over any fixed default.

Limits of a single-method default. No method dominates both mechanisms. MA-GCT leads overall (32.1%) but wins only 14.3% under MCAR; MissForest+XGB leads MCAR (54.8%) but wins only 1 of 42 MNAR cells. An always-MissForest+XGB strategy—the SBS—achieves regret 0.073, and an always-Combined-A strategy achieves regret 0.130 (Table 7). Dynamic-Learned-kNN (regret 0.083, win rate 20.5%) selects across mechanism boundaries, maintaining the highest hit rate among non-oracle strategies while avoiding the runtime penalty of always deploying graph-based methods (∼2500× slower than lightweight alternatives; Section 4.2).

**Figure 4:**
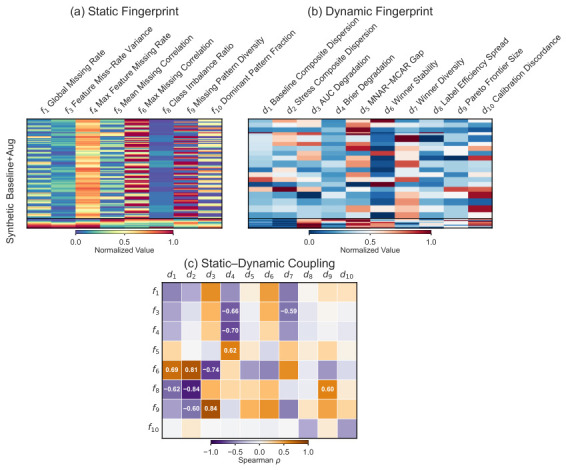
Decoupling analysis triptych. (**a**) Static fingerprint heatmap over 8 active dimensions ($f_{1}$, $f_{3}$–$f_{6}$, $f_{8}$–$f_{10}$; $f_{2}$ and $f_{7}$ are excluded as constant across datasets) for 30 base datasets and their augmented variants, min–max normalized per column; the horizontal line separates eICU-derived (above) from synthetic (below) datasets. (**b**) Dynamic fingerprint heatmap of 10 landscape descriptors ($d_{1}$–$d_{10}$), same row ordering as panel (**a**), min–max normalized per column. (**c**) Spearman rank correlation (ρ) between the 8 active static dimensions (rows) and 10 dynamic dimensions (columns); only the top 15% by |ρ| are annotated (12 of 80 cells). The sparse coupling confirms that static and dynamic fingerprints capture largely complementary dataset properties.

**Figure 5:**
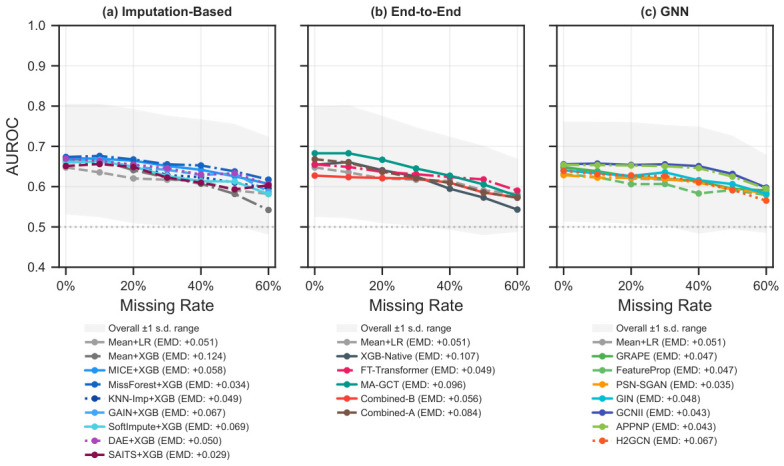
AUROC degradation curves under MCAR at 20% label rate, averaged across 20 datasets (±1 SD shaded). (**a**) Imputation-based pipelines. (**b**) End-to-end pipelines. (**c**) GNN-based pipelines. Mean+LR (dashed gray) appears in all three panels as a shared baseline. Legend entries include each method’s earth mover’s distance (EMD) from the uniform distribution.

**Figure 6:**
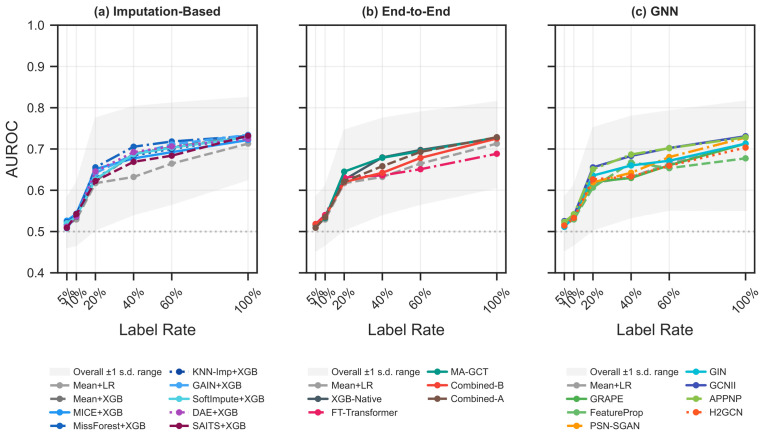
Label efficiency curves under MCAR at 30% missing rate, averaged across 20 datasets (±1 SD shaded). (**a**) Imputation-based pipelines. (**b**) End-to-end pipelines. (**c**) GNN-based pipelines. Mean+LR (dashed gray) appears in all three panels as a shared baseline.

**Figure 7:**
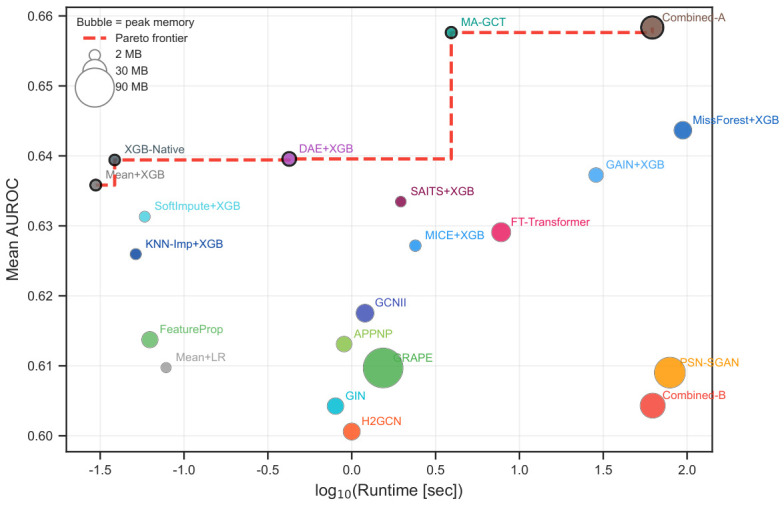
Efficiency–performance Pareto frontier. Each method is plotted by $\log_{10}$(median runtime) (*x*-axis) and mean AUROC (*y*-axis); bubble size encodes peak memory usage. The dashed red line traces the Pareto-optimal set.

Figure 4, Figure 5, Figure 6 and Figure 7 share a common analysis pipeline. (i) For each of the 20 base eICU datasets, the full degradation sweep is run: the missing rate varies from 0 to 60%, and the label rate from 5 to 100%, under both MCAR and MNAR, with all 21 methods evaluated over 30 train/test splits per condition. (ii) Static fingerprints and per-method performance vectors are extracted for each condition, feeding the decoupling analysis in Figure 4. (iii) Per-method AUROC curves are averaged across the 20 datasets to yield the degradation profiles in Figure 5 and Figure 6 (±1 SD shading shows cross-dataset variability). (iv) The Pareto frontier in Figure 7 plots mean AUROC against median runtime; bubble size encodes peak memory. All AUROC values are test-set averages with no validation set.

### 4.2. Degradation Profiles

Among the 21 methods, MissForest+XGB degrades most gently across the missingness range (Earth Mover’s Distance, EMD = 0.034), followed by PSN-SGAN (0.035) and APPNP (0.043). Mean+XGB is the most sensitive to missingness injection (EMD = 0.124), followed by XGB-Native (0.107). Combined-A (EMD = 0.084) degrades faster than its PSN-SGAN component (0.035) under MCAR, but this reverses sharply under MNAR, where Combined-A reaches the lowest EMD among all 21 methods (0.003; Supplementary Figure S1). Among lightweight GNNs, H2GCN shows the sharpest degradation beyond 40% missingness. The corresponding MNAR degradation curves (Supplementary Figure S1) show a qualitatively different ordering, with Combined-A maintaining higher AUROC at extreme injection rates, while GNN-based methods become the most sensitive (APPNP EMD = 0.134, GCNII = 0.115, H2GCN = 0.114).

At a fixed missing rate of 30% (MCAR), all methods converge to similar AUROC once labels are abundant, but diverge sharply below 20% label rate. MissForest+XGB reaches near-plateau performance by 40% labels. PSN-SGAN and Combined-A gain more steeply between 5% and 20% labels, exploiting unlabeled graph structure, yet do not overtake imputation-based pipelines at higher label rates under MCAR.

Two patterns in the degradation profiles are worth highlighting. First, method rankings reverse between mechanisms: under MCAR, MissForest+XGB holds the flattest curve (EMD = 0.034), but under MNAR it loses ground to Combined-A (MissForest+XGB’s EMD rises to 0.081 under MNAR, while Combined-A’s drops to 0.003), whose attention mechanism can encode missingness patterns directly (Supplementary Figure S1). Second, graph-based methods (PSN-SGAN, Combined-A) extract more value from the first 20% of labeled samples than imputation-based pipelines do (Figure 6), reaching near-plateau AUROC at lower label rates. These mechanism- and label-dependent ranking shifts are what make algorithm selection worthwhile: the best pipeline changes with data conditions.

Three methods form the Pareto-optimal set: Mean+XGB (AUROC 0.625, 0.01 s), XGB-Native (0.632, 0.02 s), and Combined-A (0.648, 25.2 s). Most other methods are dominated by one of these three. The large runtime gap between the lightweight end-to-end methods and Combined-A is itself a motivation for algorithm selection: the expensive Combined-A is worthwhile only when data conditions favor it (primarily under MNAR).

### 4.3. Instance Space Population

Figure 3 illustrates the augmentation effect. The 20 base datasets (all eICU, natural missingness 4–13%) cluster tightly in the low-missingness corner of the fingerprint space. After augmentation, 83 instances span the quality spectrum: MCAR-injected instances spread along $f_{1}$ (global missing rate) while maintaining low $f_{5}$ (missingness correlation); MNAR-injected instances show elevated $f_{5}$ and altered $f_{11}$ (Little’s test). The FAHZU-Kidney dataset, previously surrounded by synthetic neighbors (weighted fingerprint-space distances 0.018–0.019), now has augmented eICU instances as closer, more clinically relevant neighbors. All distances and kNN queries are computed in the weighted 10-dimensional fingerprint space, not in the t-SNE embedding; Figure 3 uses t-SNE for visualization only, and its pairwise distances are not metrically faithful.

### 4.4. Little’s Test Validation

Little’s test behaves, as shown in Table 6 as expected: heavy MCAR injection produces high *p*-values (not rejecting MCAR), consistent with the injected mechanism. MNAR-injected instances show mixed behavior—the heavy MNAR rejection rate is only 5%, because once substantial random-appearing noise is overlaid on the rank-based mechanism, the test loses power. The baseline and light MCAR conditions retain their original missingness structure (largely non-MCAR in the eICU data), showing high rejection rates. This confirms that $f_{11}$ captures meaningful mechanism information, even if it is not a perfect discriminator.

**Table 6:** Little’s MCAR test by degradation condition.

Condition	*N*	Median *p*	Mean *p*	% Reject MCAR (p<0.05)
baseline halflabel	19	0.000	0.212	79%
light MCAR	19	0.000	0.248	74%
moderate MCAR	19	1.000	0.886	11%
heavy MCAR	19	1.000	1.000	0%
moderate MNAR	19	0.832	0.615	11%
heavy MNAR	19	1.000	0.776	5%

*N* counts datasets where Little’s test was computed (all 19 eICU datasets with sufficient sample size); this differs from the per-condition instance counts in Section 4.6, which reflect sweep coverage.

### 4.5. LODO Cross-Validation

Table 7 reports the central LODO results across all eight recommender strategies.

Motivation: static kNN fails on the original store. On the original store (N=18 base datasets, baseline halflabel condition, MCAR), MA-GCT is the single best solver (SBS) with AUROC 0.745, followed by GAIN+XGB (0.738), Combined-A (0.737), and MissForest+XGB (0.732). The VBS-SBS gap is 0.038 (Oracle 0.783 − SBS 0.745). The purely static kNN (10-dim, N=18) achieves only 0.731 (regret 0.051), falling below the SBS (Δ=−0.014). With only 18 instances and uniform feature weights, static fingerprints alone cannot support effective meta-learning. The oracle gap decomposition (Section 4.7) confirms that 67% of this gap is selection regret—the recommender choosing wrong methods—rather than winner noise.

**Table 7:** Central results: base-dataset-level LODO on the augmented store (N=83, 19 base groups).

Strategy	AUROC	Regret	Win Rate
Random	0.653	0.137	4.8%
Always-GRAPE	0.658	0.132	0.0%
Always-MICE+XGB	0.684	0.106	2.4%
Always-KNN-Imp+XGB	0.683	0.107	4.8%
Always-GAIN+XGB	0.691	0.098	4.8%
Always-GCNII	0.676	0.113	4.8%
Always-Combined-A	0.660	0.130	1.2%
Always-MissForest+XGB	0.717	0.073	14.5%
Static-kNN (N=83)	0.700	0.089	15.7%
Dynamic-Learned-kNN (N=83)	0.707	0.083	20.5%
Oracle	0.790	0.000	100%

Regret = instance-level mean of (oracle AUROC − strategy AUROC). Win rate = fraction of 83 instances where the strategy’s recommendation matches the oracle-best method. All values from 21-pipeline augmented LODO.

Two contributions close this gap. On the augmented store (N=83 instances from 19 base datasets, base-dataset-level LODO), Dynamic-Learned-kNN reaches the highest win rate among non-oracle strategies (20.5% vs. 14.5% for the SBS, Always-MissForest+XGB), meaning the recommender picks the correct pipeline more often than any fixed default. Its mean regret (0.083) is, however, slightly above the SBS (0.073): when it errs, the penalty tends to exceed that of a fixed default. This asymmetry—higher hit rate but larger miss penalty—is expected for instance-adaptive selection with a small meta-instance library and a narrow VBS-SBS gap (0.073 AUROC).

The metric-learning component provides additional gains: on the same augmented store, the unweighted static kNN achieves win rate 15.7%, whereas Dynamic-Learned-kNN reaches 20.5%—a 31% relative gain attributable to the learned weights. The learned weights concentrate on $f_{1}$ (global missing rate, 47%) and $f_{9}$ (pattern diversity, 37%), consistent with the mechanism dichotomy (Section 4.7) where missing rate and structure are the strongest predictors of method identity. At deployment, the recommender requires only the 10-dimensional static fingerprint with pre-learned weights applied before kNN lookup; no sweep data is needed for new datasets.

Statistical significance: A Friedman test across 21 methods across 84 conditions confirms significant method complementarity ($\chi^{2}=674.68$, $p=5.02\times 10^{-130}$), confirming that instance-adaptive selection is worthwhile in principle. The absolute margins between recommender strategies are small, a point we return to in Section 5.2.

Regret, not raw AUROC, is the right metric for comparing strategies. The augmented evaluation tests over a wider quality range spanning six degradation conditions; so, raw AUROCs reflect condition difficulty rather than recommender quality. Regret (the gap to the per-instance oracle) normalizes for this variation.

**Figure 8:**
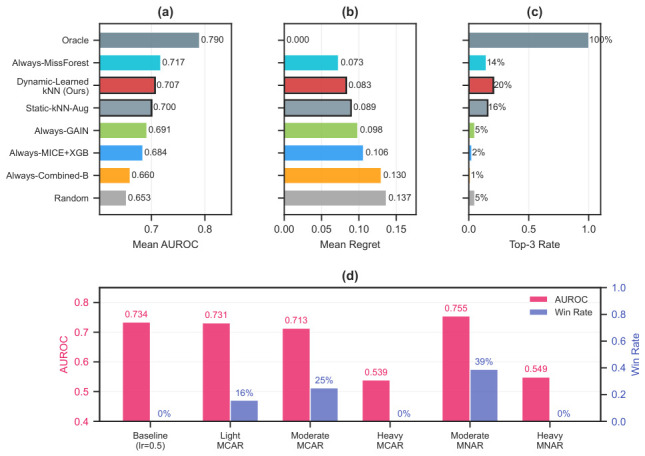
Leave-one-base-dataset-out (LODO) cross-validation on the augmented instance store (N=83). (**a**) Mean AUROC by strategy. (**b**) Mean regret (gap to per-instance oracle). (**c**) Win rate (how often the recommendation matches the oracle-best method). (**d**) Recommender AUROC and win rate broken down by degradation condition.

Dynamic-Learned-kNN has regret 0.083 on the augmented LODO track (Figure 8) comparable to the SBS (0.073). The pairwise comparison with Always-MissForest+XGB (Δ=−0.010) does not exclude zero under block bootstrap (B=2000, base datasets resampled with replacement), which is unsurprising given only 20 independent folds. Nevertheless, the recommender’s regret stays well below random selection (0.137); so, it does not degrade performance relative to an uninformed baseline.

### 4.6. Per-Degradation-Tag Analysis

Dynamic-Learned-kNN performance varies by degradation condition (Table 8).

The recommender achieves its highest AUROC (0.754) and lowest regret (0.040) on moderate MNAR conditions, where method complementarity is strongest and missingness-aware methods provide the clearest advantage. The win rate is highest under moderate MNAR (39%), where the optimal method varies most across datasets. Under baseline and heavy conditions, the win rate drops to 0%: in baseline conditions, a strong default (MissForest+XGB) typically suffices; in heavy conditions, limited coverage (N=6) and extreme degradation compress inter-method differences.

Coverage caveat: Heavy degradation conditions (miss ≥ 0.5) have limited coverage: only 30% of base datasets (N=6 each) contribute instances at these settings, because the remaining datasets lack sweep data at extreme grid points. The aggregate statistics are therefore dominated by the moderate conditions, which have near-complete coverage (80–95%).

### 4.7. Mechanism Dichotomy and Oracle Gap Analysis

**Figure 9:**
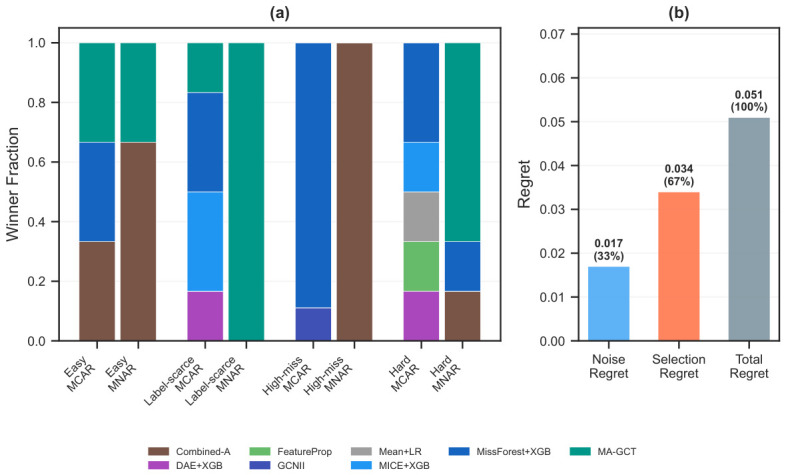
Condition-stratified analysis. (**a**) Winner fraction by regime (Easy, Label-scarce, High-miss, Hard) and mechanism (MCAR, MNAR); stacked bars show each winning method’s share. (**b**) Oracle gap decomposition: noise regret is the performance variance across repetitions; selection regret is the cost of choosing a non-optimal method.

Condition-stratified winners: We partition the 84-cell condition space into four regimes (Easy: miss ≤ 20%, label ≥ 40%; Label-scarce: miss ≤ 20%, label < 20%; High-miss: miss ≥ 40%, label ≥ 40%; Hard: miss ≥ 40%, label < 20%) and examine winners within each mechanism (Figure 9).

Under MNAR, missingness-aware methods (MA-GCT + Combined-A) jointly account for all wins in every regime. MA-GCT dominates label-scarce (100%) and hard (67%) MNAR conditions, while Combined-A leads easy (67%) and high-miss (100%) MNAR conditions. Under MCAR, winners are more dispersed, with MissForest+XGB dominating high-miss (89%). The mechanism predicts winner identity more strongly than missing rate or label rate does.

Two-rule mechanism-aware baseline: A minimal heuristic assigns the most frequent MCAR winner (MissForest+XGB) when Little’s test does not reject MCAR, and a missingness-aware method (MA-GCT or Combined-A) otherwise. On the original store (base-dataset-level LODO, N=18), most base datasets are classified as MCAR by Little’s test and thus receive MissForest+XGB (AUROC 0.732), yielding a two-rule AUROC of ≈ 0.732 with regret 0.051—essentially matching the static kNN (0.731, regret 0.051). Neither approach can exploit the full 21-method landscape: the true SBS is MA-GCT (0.745), which the two-rule heuristic assigns only to the minority of non-MCAR datasets. Both augmentation (expanding the instance library) and metric learning (weighting fingerprint dimensions) are needed to close this gap.

Causal interpretation: The mechanism dichotomy reported in Table 9 is an association between injected mechanism labels and winner identity, not evidence that the mechanism itself causally determines method performance. The injection process simultaneously alters multiple fingerprint dimensions ($f_{1}$, $f_{5}$, $f_{9}$, $f_{11}$), and these joint changes—rather than the mechanism label per se—may be the actual driver of winner shifts. In particular, MNAR injection via left-tail masking elevates inter-feature missingness correlations ($f_{5}$) and shifts pattern diversity ($f_{9}$), both of which receive substantial weight in the learned metric. The two-rule baseline works because the mechanism label is a compact proxy for this constellation of fingerprint changes, but it does not imply that knowing the mechanism alone is sufficient. For naturally occurring MNAR data—where the missingness structure may differ from our injection protocol—the two-rule heuristic is untested. The external datasets lack ground-truth mechanism labels; so, this question remains open. When a knowledge base is available, the full fingerprint-based recommender is preferable to the two-rule heuristic, because the fingerprint captures structural features that drive method performance regardless of mechanism provenance.

Oracle gap decomposition: The total gap between the oracle and the static kNN on the original store (regret = 0.051) decomposes into noise regret (0.017, 33%) and selection regret (0.034, 67%). Selection regret dominates: the recommender is choosing the wrong methods, not running up against a low ceiling. Dynamic-Learned-kNN (Section 3.9) targets this component directly; on the augmented store, it attains the highest win rate (20.5%) among non-oracle strategies (Table 7). Supplementary Figure S2 provides per-dataset breakdowns.

### 4.8. External Validation

We validated the framework on 25 external subsets across three independent cohorts (Table 1): 9 FAHZU-Kidney subsets, 8 SAHZU-CRC subsets, and 8 MIMIC-IV ICD-diagnosis groups. None were used during knowledge-base construction. The evaluation tests cross-domain generalization under eight degradation conditions per subset (varying missing rate, label rate, and mechanism).

Per-cohort method rankings are shown in Supplementary Figure S10.

Cross-domain KB-source × fingerprint ablation: A key finding of the external evaluation is that the fingerprint module must be adapted for cross-domain use. The original $f_{1}$–$f_{10}$ missingness fingerprint, designed for eICU, produces cross-domain regret of 0.066 (eICU-only KB)—worse than random selection have near-zero overlap between eICU and external datasets. Replacing the fingerprint with 6 standard pymfe meta-features [20] reduces cross-domain regret to 0.040 (eICU-only KB) or 0.025 (synthetic-only KB), the latter 55% below random. The best combination (pymfe6 + synthetic-only KB, 10 entries) achieves the lowest regret across all three external domains: CRC 0.024, KD 0.026, MIMIC-IV 0.026 (Supplementary Table S4). This demonstrates the framework’s modularity: the augmentation-and-recommendation architecture transfers across domains when the fingerprint and KB source are adapted to the target setting.

Per-domain results: Using the pymfe6 fingerprint with the eICU+synthetic KB (30 entries), cross-domain regret by domain is: CRC 0.041, KD 0.031, MIMIC-IV 0.034 (random baseline: CRC 0.074, KD 0.046, MIMIC-IV 0.045). The recommender reduces regret relative to random in all three domains. Under MNAR conditions, where the original $f_{1}$–$f_{10}$ fingerprint struggles most (regret 0.087 vs. random 0.068), pymfe6 achieves 0.040—a 41% reduction (Supplementary Table S4).

Single-condition aggregated results: On the aggregated FAHZU-Kidney cohort (N=14,164), the recommender (using $f_{1}$–$f_{10}$) selects GAIN+XGB (rank #8/21, AUROC 0.886; oracle: Mean+XGB, 0.915). The recommendation falls within the correct family (imputation-based) but loses accuracy when the query is far outside the training distribution.

On the aggregated SAHZU-CRC cohort (N=2080), the recommender selects Combined-A (rank #4/21, AUROC 0.735; oracle: MICE+XGB, 0.739)—a near-optimal choice, consistent with the query fingerprint falling inside the knowledge base’s coverage.

MIMIC-IV (8 ICD-diagnosis groups; N=1771–24,873; 28 features; 15–29% missing; US multi-center ICU [39]): Using the eICU-only KB with $f_{1}$–$f_{10}$, the recommender selects MICE+XGB for 6 of 8 groups, achieving per-group regret 0.003–0.073 (mean 0.025). The oracle method is Combined-A for five groups, Mean+XGB for two, and XGB-Native for one. MIMIC-AKI and MIMIC-Anemia subsets (N>19,000) were excluded from the sweep due to computational constraints (graph construction intractable at that scale).

These 25 external subsets suggest a practical rule: deploy the top 3 shortlist rather than a single recommendation. On FAHZU-Kidney, the oracle method (Mean+XGB) already appears in the recommended top 3; so, the top 3 regret is zero. On SAHZU-CRC, the best method in the top 3 reaches AUROC 0.735, only 0.004 below the true best (MICE+XGB, 0.739). For cross-domain deployment, switching to pymfe meta-features and/or a synthetic KB provides substantially better recommendations than the eICU-specific fingerprint (regret 0.025 vs. 0.066). When multiple fingerprint dimensions are OOD, supplement the shortlist with a targeted sweep. Supplementary Figure S5 shows the nearest-neighbour structure in fingerprint space; Section S4 gives the full OOD analysis.

## 5. Discussion

### 5.1. Instance Scarcity and Distance Quality as Dual Bottlenecks

Static meta-learning on the original store falls below the SBS (Section 4.5), because two bottlenecks compound: instance scarcity (18 datasets are too few for kNN) and distance quality (uniform feature weights). Constructive augmentation (18→83 instances) and dynamic-supervised metric learning (Section 3.9) tackle both: the augmented store gives kNN enough density, while learned weights transfer sweep-derived similarity into a static representation. On the augmented store, Dynamic-Learned-kNN attains the highest win rate (20.5%) among non-oracle strategies (Table 7), though its mean regret (0.083) is comparable to that of SBS (0.073) and confidence intervals still overlap at the current sample size. The perturbation-profiling analogy from Section 1 [22] applies here: controlled perturbations expose method-performance differences that natural missing-rate variation alone does not produce.

An ablation masking the condition-encoding dimensions ($f_{1}$, $f_{2}$, $f_{11}$) yields static-kNN regret 0.076—below the unmasked 0.089—confirming that structural dataset properties ($f_{3}$–$f_{10}$), not augmentation conditions, carry the signal (Supplementary Figure S3).

### 5.2. The VBS-SBS Gap

On the original store, the VBS-SBS gap is 0.041 AUROC; on the augmented store, it widens to 0.073 (Oracle 0.790 − SBS 0.717). Both are modest compared to SAT domains [14], partly because all methods perform reasonably on ICU data. Constructive augmentation widens the gap by introducing quality extremes where fixed baselines deteriorate, consistent with Tornede et al. [23]’s observation that narrow gaps defeat meta-learning. Oracle gap decomposition (Section 4.7) shows that selection regret accounts for most of the gap; metric learning targets this component.

Clinical significance: Per-condition regret drops to 0.040 under moderate MNAR, where method complementarity is strongest. The knowledge base and learned weights can be reused as new datasets are added without retraining.

Statistical evidence: The Friedman test ($\chi^{2}=674.68$, $p=5.02\times 10^{-130}$, 21 methods across 84 conditions) confirms significant method complementarity. On the augmented store, Dynamic-Learned-kNN has the highest win rate among non-oracle strategies (20.5%), while its mean regret (0.083) is comparable to the SBS (0.073). Cross-domain validation on 25 external subsets provides independent evidence: pymfe6 + synthetic-only KB achieves regret 0.025, 55% below random (Section 4.8). Expanding the dataset library remains the most direct route to narrowing within-domain confidence intervals.

Failure cases: The recommender underperforms on datasets where rare-winner methods (e.g., GAIN+XGB, <5% global wins) are locally optimal—the kNN frequency vote systematically underrecommends them. eicu-12 (TBI) exemplifies this with regret 0.069. Failures concentrate where rare winners are uniquely suited or knowledge-base coverage is thin; expanding the knowledge base would mitigate both.

### 5.3. Little’s Test as Mechanism Indicator

$f_{11}$ behaves as expected for MCAR: heavy injection (30–50%) produces high *p*-values (0–11% rejection). For MNAR, heavy injection shows only 5% rejection, because Little’s joint test lacks power to detect column-wise non-randomness overlaid on substantial existing missingness [21]. Baseline conditions (0–10% MCAR injection) show 74–79% rejection, reflecting the non-MCAR nature of native eICU missingness.

What matters is not whether $f_{11}$ classifies mechanisms correctly, but whether it separates instances in a way that predicts method performance. The 39% win rate under moderate MNAR suggests it does [17,20], though part of that rate reflects winner concentration among a few missingness-aware architectures rather than fine-grained discrimination.

MAR implications: MAR is covered through two routes. First, native eICU missingness already contains a substantial MAR component (74–79% MCAR rejection rate; Table 6), reflecting diagnosis-conditional ordering practices. Second, synthetic instances generated via a conditional probability chain (diagnosis→procedure→lab result) are MAR by construction: a lab value is missing because the corresponding procedure was not ordered given the diagnosis—an observed variable—not because of the missing value itself. Cross-domain validation on MIMIC-IV—whose missingness is predominantly MAR—provides a direct test: the recommender achieves mean regret 0.025 across 8 ICD groups (Section 4.8), confirming that the framework generalizes to MAR-dominant settings. In the fingerprint space, $f_{5}$ (missingness correlation) and $f_{9}$ (pattern diversity) capture the inter-variable dependencies that separate MAR from MCAR.

### 5.4. Practical Guidance

Target scenario: The framework is designed for clinical groups that have few datasets and cannot afford exhaustive method sweeps. It complements per-dataset AutoML [18]: the top *k* recommended methods can warm-start a targeted search, narrowing the candidate space from 21 methods to 3–5.

Deployment: Compute the 10-dimensional fingerprint (<1 s), apply learned weights, query kNN (ms): under 2 s total, no model training required. For moderate conditions (missing < 30%, labels > 20%), MICE+XGB is a strong default; graph methods add value mainly at quality extremes. When no knowledge base is available, the mechanism-aware fallback (MCAR→MissForest+XGB, non-MCAR→Combined-A via Little’s test) outperforms the uninformed static kNN (Section 4.7).

When to distrust: if the query fingerprint is OOD on ≥3 dimensions or the nearest-neighbor distance exceeds the 95th percentile of stored pairwise distances, validate with a targeted top 3 sweep.

Computational cost: Using the released knowledge base incurs zero sweep cost. Building a local KB requires ~100 GPU-hours on a single NVIDIA RTX 3090 (~100 wall-clock hours; one-time), reducible by sweeping only promising methods identified by the pre-built KB.

Missing trial endpoints: When primary outcomes or safety variables are missing, the recommended workflow is: compute the fingerprint on available data, query the recommender for a top 3 shortlist, and check whether the fingerprint falls inside the training distribution. If $f_{1}>0.6$ or $f_{2}<0.05$, the OOD safeguard (Section 4.8) applies and a targeted sweep should precede deployment. For regulatory-grade analyses, the shortlist should be treated as a starting point, supplemented by sensitivity analyses (e.g., pattern-mixture models or tipping-point analyses) required by the relevant clinical trial guidelines.

### 5.5. Limitations

The primary limitation is source homogeneity. All 20 base datasets share eICU’s 27-variable schema, and augmented instances derive from this single source using one perturbation protocol. While the shared schema controls feature-level confounding, the learned weights and winner map may encode eICU-specific relationships that do not transfer to datasets with different variable compositions, higher dimensionality, or wider native missing-rate ranges. External validation on 25 subsets across three independent cohorts (8 CRC, 9 KD, 8 MIMIC-IV) provides cross-schema, cross-institution, and cross-country evidence. The cross-domain fingerprint ablation (Section 4.8) reveals that adapting the fingerprint module (pymfe6 instead of $f_{1}$–$f_{10}$) is necessary for cross-domain transfer (regret 0.025 vs. 0.066). All validation data comes from hospital settings; ambulatory EHR and longitudinal follow-up remain untested. Additionally, MNAR injection uses only left-tail masking, reflecting clinical patterns where low values are selectively unrecorded. Other MNAR variants—right-tail truncation (capped sensor readings), trajectory-dependent dropout (sicker patients leaving this study), threshold-dependent censoring—are not modeled. That said, the knowledge base now spans three structurally distinct mechanisms (MCAR injection, left-tail MNAR injection, MAR via synthetic conditional-probability chains), and the fingerprint encodes structural properties rather than mechanism labels, which may confer partial robustness to unseen variants.

Several methodological constraints affect the evaluation. LODO yields only ∼19 test folds, constraining statistical power for strategy comparisons. Of 120 candidate augmentations, 37 are excluded due to insufficient coverage, and extreme degradation conditions (miss ≥ 0.5) cover only 32% of base datasets. The learned weights assume linear feature interactions; non-linear effects are not captured. All 21 methods use fixed hyperparameters without per-dataset tuning, matching clinical settings where per-method optimization is rarely feasible; this may disadvantage hyperparameter-sensitive GNN methods (baselines from 2019–2020) relative to simpler pipelines whose defaults transfer better across datasets; even so, missingness-aware methods (MA-GCT + Combined-A) jointly win 41 of 42 MNAR cells (97.6%), confirming that fixed defaults do not systematically exclude complex methods. AUROC is the sole evaluation metric; AUPRC and Brier score may yield different winner maps (Supplementary Figure S4). The fingerprint dimensions were selected on conceptual grounds without formal feature-selection ablation.

Finally, two datasets (eicu-18, eicu-19) yield near-chance AUROC across all methods, contributing zero VBS-SBS gap; they are retained to avoid biasing the meta-analysis but reduce overall performance statistics. External validation on 25 subsets across three cohorts (CRC, KD, MIMIC-IV) provides cross-domain evidence, but does not establish generalization to arbitrary clinical settings; we recommend local targeted sweeps when ≥2 fingerprint dimensions fall outside the training range.

## 6. Conclusions

We proposed constructive instance augmentation and dynamic-supervised metric learning for algorithm selection in clinical prediction. Three findings stand out. First, augmentation matters: on the original store (N=20), static meta-learning cannot beat the single best solver, but on the augmented store (N=83), Dynamic-Learned-kNN attains the highest win rate (20.5%) among non-oracle strategies—it picks the right pipeline more often than any fixed default, although its mean regret remains comparable to that of SBS under the narrow VBS-SBS gap (0.073 AUROC). Second, the learned weights concentrate on the global missing rate (47%) and pattern diversity (37%), the two dimensions most aligned with the MCAR-vs-MNAR mechanism dichotomy: missingness-aware methods (MA-GCT + Combined-A) jointly win 41 of 42 MNAR cells (97.6%), while MissForest+XGB dominates MCAR (23/42, 54.8%). Third, cross-domain evaluation on 25 external subsets (8 CRC, 9 KD, 8 MIMIC-IV) demonstrates that the framework generalizes when the fingerprint module is adapted: pymfe6 + synthetic-only KB achieves regret 0.025, 55% below random selection, whereas the eICU-specific $f_{1}$–$f_{10}$ fingerprint does not transfer (regret 0.066, worse than random). This highlights the framework’s modularity—its value lies in the augmentation-and-recommendation architecture, not in a particular feature set. Several gaps remain: (a) base datasets share eICU’s schema, though external validation spans 18–28 features across three specialties and two countries; (b) MNAR simulation covers only left-tail masking, while MAR is represented via synthetic instances; (c) roughly 20 LODO folds limit within-domain statistical power; (d) AUROC is the sole evaluation metric; and (e) ambulatory and longitudinal EHR settings are untested. Multi-source benchmarks, broader augmentation protocols, and a larger meta-instance library are the most direct next steps. We release the benchmarking framework, fingerprint definitions, and augmentation protocol as open infrastructure at https://github.com/xiaolibird/MissingFPS.

## Figures and Tables

**Table 1:** Dataset overview.

Source	ID	*N*	*F*	Miss. (%)	Description
eICU	eicu-00	2083	27	7.3	MICU sepsis
eICU	eicu-01	647	27	9.2	MICU pneumonia
eICU	eicu-02	408	27	11.0	MICU COPD
eICU	eicu-03	1060	27	9.4	SICU trauma
eICU	eicu-04	1501	27	7.1	SICU gastrointestinal
eICU	eicu-05	410	27	6.7	SICU renal
eICU	eicu-06	1926	27	11.7	CCU arrhythmia
eICU	eicu-07	1033	27	10.0	CCU CHF
eICU	eicu-08	2590	27	9.2	CCU AMI
eICU	eicu-09	5064	27	8.3	CSRU cardiac surgery
eICU	eicu-10	12,107	27	9.5	CSRU all postoperative
eICU	eicu-11	3509	27	12.7	NSICU stroke
eICU	eicu-12	832	27	11.3	NSICU traumatic brain injury
eICU	eicu-13	14,934	27	10.0	MICU all large-A
eICU	eicu-14	14,934	27	9.6	MICU all large-B
eICU	eicu-15	3968	27	7.1	CCU+MICU sepsis
eICU	eicu-16	40,165	27	9.8	CCU+MICU all
eICU	eicu-17	216	27	6.0	MICU+SICU hepatic failure
eICU	eicu-18	76	27	4.2	SICU pancreatitis
eICU	eicu-19	571	27	11.7	NSICU seizure
External	FAHZU-Kidney	14,164	28	43.7	Hemodialysis, 1-year mortality
External	SAHZU-CRC	2080	18	5.5	Colorectal cancer, 5-year survival
External	MIMIC-Circ	24,873	28	24.4	Circulatory diseases
External	MIMIC-Dig	5793	28	19.3	Digestive diseases
External	MIMIC-Endo	1798	28	25.4	Endocrine disorders
External	MIMIC-Inf	6673	28	15.2	Infectious diseases
External	MIMIC-Inj	9747	28	27.2	Injury and poisoning
External	MIMIC-Neo	5475	28	26.5	Neoplasms
External	MIMIC-Nerv	1771	28	29.2	Nervous system diseases
External	MIMIC-Resp	3967	28	23.8	Respiratory diseases

*N* = number of patients; *F* = number of features; Miss. = native missing rate before any injection.

**Table 2:** Twenty-one candidate methods.

#	Method	Paradigm	Reference
1	Mean+LR	Impute+Classify	baseline
2	Mean+XGB	Impute+Classify	baseline
3	MICE+XGB	Impute+Classify	[40]
4	MissForest+XGB	Impute+Classify	[41]
5	KNN-Imp+XGB	Impute+Classify	—
6	GAIN+XGB	Impute+Classify	[42]
7	SoftImpute+XGB	Impute+Classify	[43]
8	DAE+XGB	Impute+Classify	[44]
9	SAITS+XGB	Impute+Classify	[45]
10	GRAPE	Impute+Classify	[4]
11	FeatureProp	Impute+Classify	[46]
12	PSN-SGAN	Impute+Classify	[47]
13	Combined-A (GCT→PSN-SGAN)	Impute+Classify	this work
14	Combined-B (PSN-SGAN→GCT)	Impute+Classify	this work
15	GIN	Impute+Classify	[6]
16	GCNII	Impute+Classify	[7]
17	APPNP	Impute+Classify	[31]
18	H2GCN	Impute+Classify	[8]
19	XGB-Native	End-to-End	[48]
20	FT-Transformer	End-to-End	[49]
21	MA-GCT	End-to-End	[5]

**Table 3:** Eleven-dimensional missingness fingerprint.

Dim	Name	Description	Range
$f_{1}$	global missing rate	$1-\bar{M}$, where $\bar{M}$ is the mean observation rate across the data matrix	[0, 1]
$f_{2}$	label rate	(y≠−1)/N	[0, 1]
$f_{3}$	feature miss variance	var (per-feature miss)/0.25	[0, 1]
$f_{4}$	max feature missing	max per-feature miss rate	[0, 1]
$f_{5}$	mean miss correlation	mean $\left|\mathrm{corr}(M_{i},M_{j})\right|$ off-diagonal	[0, 1]
$f_{6}$	max miss correlation	max $\left|\mathrm{corr}(M_{i},M_{j})\right|$ off-diagonal	[0, 1]
$f_{7}$	missing label corr	|corr(rowmiss,label)|	[0, 1]
$f_{8}$	class imbalance	min class/max class	[0, 1]
$f_{9}$	pattern diversity	unique missing patterns/*N*	[0, 1]
$f_{10}$	dominant pattern	most common pattern/*N*	[0, 1]
$f_{11}$	Little’s MCAR test	$-\log_{10}\left(p\right)$ of Little’s test [21] ^†^	≥0

^†^ Mechanism indicator only; $f_{11}$ is retained for analysis (Section 5.3) but does not enter kNN distance computation (Section 3.9). The recommender uses $f_{1}$–$f_{10}$ with learned weights.

**Table 4:** Augmentation grid (six conditions per base dataset).

Tag	Miss. Injected	Label Rate	Mechanism
baseline halflabel	0%	50%	natural
light MCAR	10%	60%	MCAR
moderate MCAR	30%	20%	MCAR
heavy MCAR	50%	10%	MCAR
moderate MNAR	20%	40%	MNAR
heavy MNAR	40%	10%	MNAR

**Table 5:** Recommender strategies evaluated.

Strategy	Store	Description
Random	both	Uniform random method selection
Always-MICE+XGB	both	Always recommend MICE+XGB
Always-MissForest+XGB	both	Always recommend MissForest+XGB
Always-GAIN+XGB	both	Always recommend GAIN+XGB (SBS on original store)
Always-Combined-A	both	Always recommend Combined-A
Static-kNN	orig	Weighted kNN on 10-dim FP (N=18)
Dynamic-Learned-kNN	aug	Metric-learned kNN on 10-dim FP; weights via DE against dynamic distances (N=83; Section 3.9)
Oracle	both	Upper bound (best method per instance)

Store: “both” = evaluated on both the original (N=20) and augmented (N=83) stores; “orig” = original only; “aug” = augmented only. Static-kNN uses the original store with uniform feature weights. Dynamic-Learned-kNN uses the augmented store, where the larger instance library makes metric learning feasible.

**Table 8:** Per-degradation-tag performance of Dynamic-Learned-kNN (augmented LODO, N=83).

Tag	Mean AUROC	Regret	Win Rate	*N*	Coverage
moderate MNAR	0.754	0.040	39%	18	90%
baseline halflabel	0.734	0.068	0%	18	90%
light MCAR	0.731	0.089	16%	19	95%
moderate MCAR	0.713	0.093	25%	16	80%
heavy MNAR	0.549	0.132	0%	6	30%
heavy MCAR	0.539	0.161	0%	6	30%

**Table 9:** Regime × mechanism winner analysis (21 methods).

Regime	MCAR Top Winner	MNAR Top Winner
Easy (9 conds each)	3-way tie: MissForest/MA-GCT/Combined-A (33% each)	Combined-A (67%)
Label-scarce (6 each)	MissForest/MICE+XGB (33% each)	MA-GCT (100%)
High-miss (9 each)	MissForest+XGB (89%)	Combined-A (100%)
Hard (6 each)	MissForest+XGB (33%)	MA-GCT (67%)

## Data Availability

The eICU-CRD v2.0 data presented in this study are available in PhysioNet at https://physionet.org/content/eicu-crd/2.0/ [38]. The MIMIC-IV data are available in PhysioNet at https://physionet.org/content/mimiciv/3.1/ [53]. The FAHZU-Kidney and SAHZU-CRC datasets are not publicly available due to hospital regulations and patient confidentiality, but are available on request from the corresponding author, Y.T. (tyler@zju.edu.cn).

## Supplementary Materials

The following supporting information can be downloaded at: https://www.mdpi.com/article/10.3390/bioengineering13050497/s1. Section S1 (detailed architecture and training procedure for PSN-SGAN, Combined-A, and Combined-B); Section S2 (hyperparameter settings for all 21 pipelines, including SAITS+XGB and MA-GCT; Table S1, hyperparameter settings for all 21 candidate pipelines); Section S3 (supplementary figures: Figure S1, MNAR performance profiles—AUROC degradation (panels (a)–(c), at 20% label rate) and label efficiency (panels (d)–(f), at 30% missing rate) under MNAR, organized by pipeline family (imputation-based, end-to-end, GNN-based); Figure S2, per-dataset oracle gap decomposition; Figure S3, LODO ablation with condition-masked fingerprints; Figure S4, LODO-CV Brier score and Brier regret; Figure S5, nearest-neighbour recommendation for external validation cohorts in fingerprint space; Figure S6, bootstrap 95% confidence intervals on strategy regret; Figure S7, method top-*k* hit rates across the full condition grid; Figure S8, MCAR vs. MNAR win distribution for missingness-aware methods; Figure S9, fingerprint-space t-SNE visualization; Figure S10, external validation per-method AUROC rankings); Table S2 (external dataset OOD analysis: FAHZU-Kidney and SAHZU-CRC); Table S3 (external dataset OOD analysis: MIMIC-IV across 8 ICD-diagnosis groups); Table S4 (KB-source × fingerprint × mechanism ablation on 25 cross-domain subsets); Table S5 (MIMIC-IV per-group recommender results, eICU-only KB, $f_{1}$–$f_{10}$ fingerprint); Section S4 (external validation details: OOD analysis and per-cohort discussion).

## Abbreviations

The following abbreviations are used in this manuscript:

APPNP	Approximate Personalized Propagation of Neural Predictions
ASP	Algorithm Selection Problem
AUROC	Area Under the Receiver Operating Characteristic Curve
DAE	Denoising Autoencoder
DE	Differential Evolution
EHR	Electronic Health Record
EMD	Earth Mover’s Distance
GAIN	Generative Adversarial Imputation Network
GCS	Glasgow Coma Scale
GCT	Graph Convolutional Transformer
GCNII	Graph Convolutional Network with Initial residual and Identity mapping
GIN	Graph Isomorphism Network
GNN	Graph Neural Network
GRAPE	Graph Partial Estimation
H2GCN	Heterophily-aware Graph Convolutional Network
ISA	Instance Space Analysis
kNN	k-Nearest Neighbors
LR	Logistic Regression
LODO	Leave-One-Dataset-Out
MAR	Missing At Random
MCAR	Missing Completely At Random
MICE	Multivariate Imputation by Chained Equations
MLP	Multilayer Perceptron
MNAR	Missing Not At Random
OOD	Out-of-Distribution
PSN	Patient Similarity Network
SAITS	Self-Attention-based Imputation for Time Series
SD	Standard Deviation
SBS	Single Best Solver
t-SNE	t-Distributed Stochastic Neighbor Embedding
VBS	Virtual Best Solver
WGAN-GP	Wasserstein GAN with Gradient Penalty
XGB	Extreme Gradient Boosting
