# Supplementary material for: Prediction Pipeline Selection for Incomplete Clinical Data via Missingness Fingerprints and Instance Augmentation
Source: Bioengineering (Basel). 2026 Apr 24;13(5):497. doi: 10.3390/bioengineering13050497 (PMC13203696; doi:10.3390/bioengineering13050497)
Supplement: Supplementary file 1 [file bioengineering-13-00497-s001.zip › bioengineering-4240940-supplementary.pdf]

Supplementary Material

Prediction Pipeline Selection for Incomplete Clinical Data via Missingness Fingerprints and Instance Augmentation

Section S1. Detailed Architecture and Training Procedure for Novel Methods

Three of the twenty-one candidate pipelines—PSN-SGAN, Combined-A, and Combined-B—are contributed by this work (or adapted without modification from our prior implementation [1]). This section gives the full architectural and training details for reproducibility. All hyperparameters are fixed across all 20 eICU subsets and all degradation conditions; no per-dataset tuning is performed.

S1.1 PSN-SGAN: Patient Similarity Network with Semi-Supervised GAN

Preprocessing. Missing values are imputed with per-column training-set means, followed by z-score standardization (fitted on training data only). Both training and test samples are preprocessed identically.

Graph construction. A k-nearest-neighbor graph (k=30, Euclidean distance) is built over the standardized feature matrix combining training and test samples (transductive setting). Edge weights are Jaccard similarities computed on neighbourhood overlap: for each pair of connected nodes i, j, the weight is $|N(i) \cap N(j)|/|N(i) \cup N(j)|$, where $N(\cdot)$ denotes the k-NN set. Edges with zero Jaccard weight are removed. Isolated nodes receive a self-loop as fallback.

Node embeddings. Node2Vec embeddings are generated on the Jaccard-weighted k-NN graph (random seed 42, single worker) and concatenated with the standardized features as network input. Generator, discriminator, and WGAN-GP architectures are specified in Section 3.3 of the main text.

Training. The model is trained with the WGAN-GP objective (gradient penalty λ_{gp}=10). The discriminator is updated n_{critic}=5 steps per generator step, and generator parameters use an exponential moving average (EMA, decay = 0.999). Training uses Adam with learning rate 3×10^{-3} and momentum $\beta_1 = 0.5$. Batch size is $\min(32, n_{\text{labelled}})$. Early stopping with patience 20 epochs is applied.

Inference. Test-node probabilities are obtained by forward-passing the concatenated features and Node2Vec embeddings through the trained discriminator, followed by softmax normalization.

Fallback. On training failure (e.g., degenerate splits with < 4 labelled samples), the pipeline falls back to MICE+XGBoost.

S1.2 GCT: Graph Convolutional Transformer Module

The GCT encoder is used as a shared component by the standalone Missing-GCT pipeline and both Combined methods. We describe it once here.

Feature embedding. Architecture details (per-feature projections, mask embeddings, hidden dimension H=64) are given in Section 3.3 of the main text. Feature types (continuous,

binary, ordinal) are auto-inferred from the training data; continuous features are z-score normalized, while binary and ordinal features retain their integer values.

Guide matrix. The absolute-value pairwise Pearson correlation matrix $|\mathbf{R}|$ is computed from the training data using pairwise-complete observations (minimum 10 paired observations per entry; otherwise the entry defaults to 0). This matrix guides the attention mechanism: entries with $|r| > 0.1$ enable attention between the corresponding feature positions, and the row-normalized correlation serves as a prior for the first attention layer.

Transformer. The feed-forward sub-layer within each transformer layer uses expansion factor 2 and dropout 0.1. KL regularization (weight = 0.1) between consecutive-layer attention distributions is applied during training. All other architectural details (number of layers, CLS token, correlation-guided attention) are specified in Section 3.3.

Classification. Training uses Adam (learning rate 10^{-3}, cosine annealing schedule, gradient clipping $\|\cdot\|_{\max}$=1.0) with binary cross-entropy loss on labelled samples only.

S1.3 Combined-A (GCT → PSN-SGAN)

The two-stage ordering (GCT→PSN-SGAN) is described in Section 3.3 of the main text. This section provides training-specific details.

Stage 1: GCT encoding. GCT is trained on labelled training samples for up to 20 epochs with early stopping (patience 20), using Adam (lr 10^{-3}, cosine annealing, gradient clipping $\|\cdot\|_{\max}$=1.0) and binary cross-entropy plus KL attention regularization (weight 0.1). After training, `[CLS]` hidden states are extracted for *all* samples (both training and test), producing 64-dimensional embeddings. These embeddings are fully observed (no missing values).

Stage 2: PSN-SGAN classification. The 64-dimensional CLS embeddings replace the raw features as input to PSN-SGAN. The k-NN graph, Node2Vec embeddings, generator, discriminator, and training procedure are identical to standalone PSN-SGAN (Section S1.1), except that the input dimension is $64 + 64 = 128$ (CLS embedding + Node2Vec embedding) instead of $F + 64$. No observation mask is needed because the CLS embeddings are fully observed.

Fallback. MICE+XGBoost on the original features if either stage fails.

S1.4 Combined-B (PSN-SGAN → GCT)

The two-stage ordering (PSN-SGAN→GCT) is described in Section 3.3. This section provides training-specific details.

Stage 1: PSN-SGAN pseudo-labelling. PSN-SGAN is trained on the raw features with the available labels (same procedure as Section S1.1). The trained discriminator is then applied to all unlabelled training samples. Predictions with $\max_c p(c) \geq 0.8$ (confidence threshold) are accepted as pseudo-labels; lower-confidence predictions are discarded.

Stage 2: GCT with augmented supervision. The original labels are augmented with the accepted pseudo-labels. GCT is trained on this larger label set for up to 50 epochs with early stopping (patience 20), using the same optimizer and loss as Section S1.2. The higher epoch budget (50 vs. 20 for Combined-A) accounts for the larger effective training set. All other GCT settings are identical to Section S1.2.

Fallback. If Stage 1 fails, the method falls back to standalone GCT. If Stage 2 also fails, MICE+XGBoost is used.

Note on code–paper consistency. The hyperparameter values reported above (and in Table S1) are extracted directly from the implementation code. One PSN-SGAN value differs from the original paper [1]: k=30 (vs. k=20 in the original), reflecting the eICU cohort structure where a wider neighbourhood improves Jaccard-weighted graph quality. All other architecture dimensions (z_{dim}=200, generator and discriminator layer sizes) follow the original. The GCT learning rate is 10^{-3} (code default), consistent with our implementation.

Section S2. Hyperparameter Settings for All Twenty-One Pipelines

Table S1 lists the key hyperparameters for all twenty-one candidate methods. All values are fixed defaults applied uniformly across all 20 eICU subsets and all degradation conditions. No per-dataset hyperparameter tuning is performed; hyperparameters use either the values recommended in the original publications or sensible defaults for the eICU cohort scale. The "Source" column indicates whether each setting follows the original paper's recommendation or is an implementation default.

Table S1: Hyperparameter settings for all twenty-one candidate pipelines. "Orig." = original paper default; "Impl." = implementation default for this benchmark.

#	Method	Key Hyperparameters	Source
1	Mean+LR	Imputer: column mean. Classifier: LogisticRegression, C=1.0, solver=saga, max_iter = 1000	Impl. (sklearn default C)
2	Mean+XGB	Imputer: column mean. Classifier: XGBoost, n_est = 200, depth = 4, lr = 0.1, subsample = 0.8, colsample = 0.8, loss=logloss	Impl.
3	MICE+XGB	Imputer: MICE, max_iter = 5. Classifier: XGBoost, n_est = 100, depth = 4, lr = 0.1, loss=logloss	Orig. (van Buuren & Groothuis-Oudshoorn, 2011)
4	MissForest+XGB	Imputer: MissForest, RF n_est = 100, max_iter = 10. Classifier: XGBoost, n_est = 100, depth = 6, lr = 0.1	Orig. (Stekhoven & Bühlmann, 2012)
5	KNN-Imp+XGB	Imputer: KNNImputer, k=5. Classifier: XGBoost, n_est = 100, depth = 6, lr = 0.1	Impl. (sklearn default k)
6	GAIN+XGB	Imputer: GAIN, G/D hidden = [256,256], hint_rate = 0.9, α=100, batch = 128, lr = 0.001, iters = 5000, optim=Adam. Classifier: XGBoost, n_est = 100, depth = 6, lr = 0.1	Orig. (Yoon et al., 2018)
7	SoftImpute+XGB	Imputer: SoftImpute, max_rank = 20, λ=1.0, max_iter = 50. Classifier: XGBoost, n_est = 100, depth = 6, lr = 0.1	Orig. (Mazumder et al., 2010)

(continued on next page)

(continued from previous page)

#	Method	Key Hyperparameters	Source
8	DAE+XGB	Imputer: Denoising AE, enc=[256,128], dec=[256,F], corruption = 0.2, lr = 0.001, batch = 128, epochs = 200, optim=Adam, loss=MSE. Classifier: XGBoost, n_est = 100, depth = 6, lr = 0.1	Orig. (Gondara & Wang, 2018)
9	SAITS+XGB	Imputer: SAITS attention, d_{model}=64, heads = 4, d_{ffn}=128, layers = 2, ORT + MIT loss (weight = 1.0), lr = 0.001, batch = 64, epochs = 100, optim=Adam. Classifier: XGBoost, n_est = 100, depth = 6, lr = 0.1	Orig. (Du et al., 2023)
10	GRAPE	Bipartite GNN, hidden = 64, layers = 2, lr = 0.001, epochs = 30, optim=Adam (wd = 1e-4), loss=MSE (edge recon.). Classifier: LR (C=1.0, max_iter=500)	Orig. (You et al., 2020)
11	FeatureProp	KNN graph (k=10), propagation iters = 40, partial-distance kernel. Classifier: LR (max_iter=1000)	Orig. (Rossi et al., 2022)
12	PSN-SGAN†	KNN graph (k=30, Euclidean), Jaccard edge weights. Node2Vec embed dim = 64. Gen: z=200 $\rightarrow$ $[500, 500]$ $\rightarrow$ F+64, batch normalization. Disc: $[500, 500, 250, 250, 250]$, weight-normalized. WGAN-GP, λ_{gp}=10, n_{critic}=5, EMA = 0.999. lr = 0.003, β_1=0.5, batch = $\min(32, n_{\text{lab}})$, early stopping (patience 20)	Adapted from Li et al. (2023); k=30 (impl.)
13	Combined-A† (GCT$\rightarrow$PSN-SGAN)	Stage 1: GCT encoding, hidden = 64, max epochs = 20, lr = 10^{-3}, cosine anneal., grad clip = 1.0, early stop (patience 20), 2 transformer layers. Stage 2: PSN-SGAN on 64-d CLS embeddings. (same as #12 but input = 128)	Impl. (this work); GCT from Choi et al. (2020), SGAN from Li et al. (2023)

(continued on next page)

(continued from previous page)

#	Method	Key Hyperparameters	Source
14	Combined-B† (PSN-SGAN→GCT)	Stage 1: PSN-SGAN pseudo-labels, (same as #12).<br>conf. threshold = 0.8.<br>Stage 2: GCT with augmented labels,<br>hidden = 64, max epochs = 50,<br>lr = 10^{-3}, cosine anneal.,<br>grad clip = 1.0,<br>early stop (patience 20),<br>2 transformer layers	Impl. (this work); threshold 0.8 from PSN-SGAN default
15	GIN	KNN graph (k=10, Euclidean),<br>hidden = 64, layers = 3,<br>dropout = 0.5, epochs = 30,<br>lr = 0.01, optim=Adam (wd = 5e-4), loss=CE.<br>Preproc: mean impute + scale	Orig. (Xu et al., 2019)
16	GCNII	KNN graph (k=10),<br>hidden = 64, layers = 8,<br>α=0.1, θ=0.5,<br>dropout = 0.5, epochs = 30,<br>lr = 0.01, optim=Adam (wd = 5e-4), loss=CE.<br>Preproc: mean impute + scale	Orig. (Chen et al., 2020)
17	APPNP	KNN graph (k=10),<br>hidden = 64, K_{diff}=10,<br>α_{teleport}=0.1,<br>epochs = 30, lr = 0.01,<br>optim=Adam (wd = 5e-4), loss=CE.<br>Preproc: mean impute + scale	Orig. (Gasteiger et al., 2019)
18	H2GCN	KNN graph (k=10),<br>hidden = 64, 3-way concat (ego + 1-hop + 2-hop),<br>dropout = 0.5, epochs = 30,<br>lr = 0.01, optim=Adam (wd = 5e-4), loss=CE.<br>Preproc: mean impute + scale	Orig. (Zhu et al., 2020)
19	XGB-Native	XGBoost with native NaN handling,<br>n_est = 200, depth = 6,<br>lr = 0.1, loss=logloss.<br>No imputation	Orig. (Chen & Guestrin, 2016)

(continued on next page)

(continued from previous page)

#	Method	Key Hyperparameters	Source
20	FT-Transformer	d_{token}=64, layers = 3, heads = 4, ffn = $4 \times d$, dropout = 0.2, learnable [MASK] embed. lr = 0.001, batch = 128, optim=Adam (wd = 1e-5), epochs = 30, loss=CE	Orig. (Gorishniy et al., 2021)
21	MA-GCT	Missing-aware GCT, hidden = 64, 2 transformer layers, 2 feedforward blocks, ffn dropout = 0.1, guide: $\lvert r_{\text{Pearson}} \rvert$, thresh = 0.1, learnable [MASK] embed. lr = 0.001, epochs = 20, optim=Adam, loss=CE. Fallback: MICE+XGB	Impl.; GCT from Choi et al. (2020)

† Methods contributed by this work (see Section S1 for full architectural details).

Abbreviations. n_est = number of estimators/trees; depth = max tree depth; lr = learning rate; wd = weight decay; CE = cross-entropy; LR = logistic regression; F = number of input features; n_{lab} = number of labelled training samples.

Shared settings. All XGBoost classifiers use `eval_metric=logloss` and `use_label_encoder=False`. All graph-based methods (GIN, GCNII, APPNP, H2GCN, PSN-SGAN) operate in a transductive setting where test nodes are present in the graph during training but their labels are withheld. Random seeds are controlled per repetition (up to 30 independent train/test splits).

Section S3. Supplementary Figures

This section collects ten supplementary figures that complement the main text: MNAR performance profiles (degradation and label efficiency), per-dataset oracle gap decomposition, recommender ablation, Brier-score evaluation, external validation nearest-neighbour visualization, bootstrap regret analysis, top-k hit rates, MCAR/MNAR win distribution, fingerprint-space t-SNE visualization, and external validation comparison.

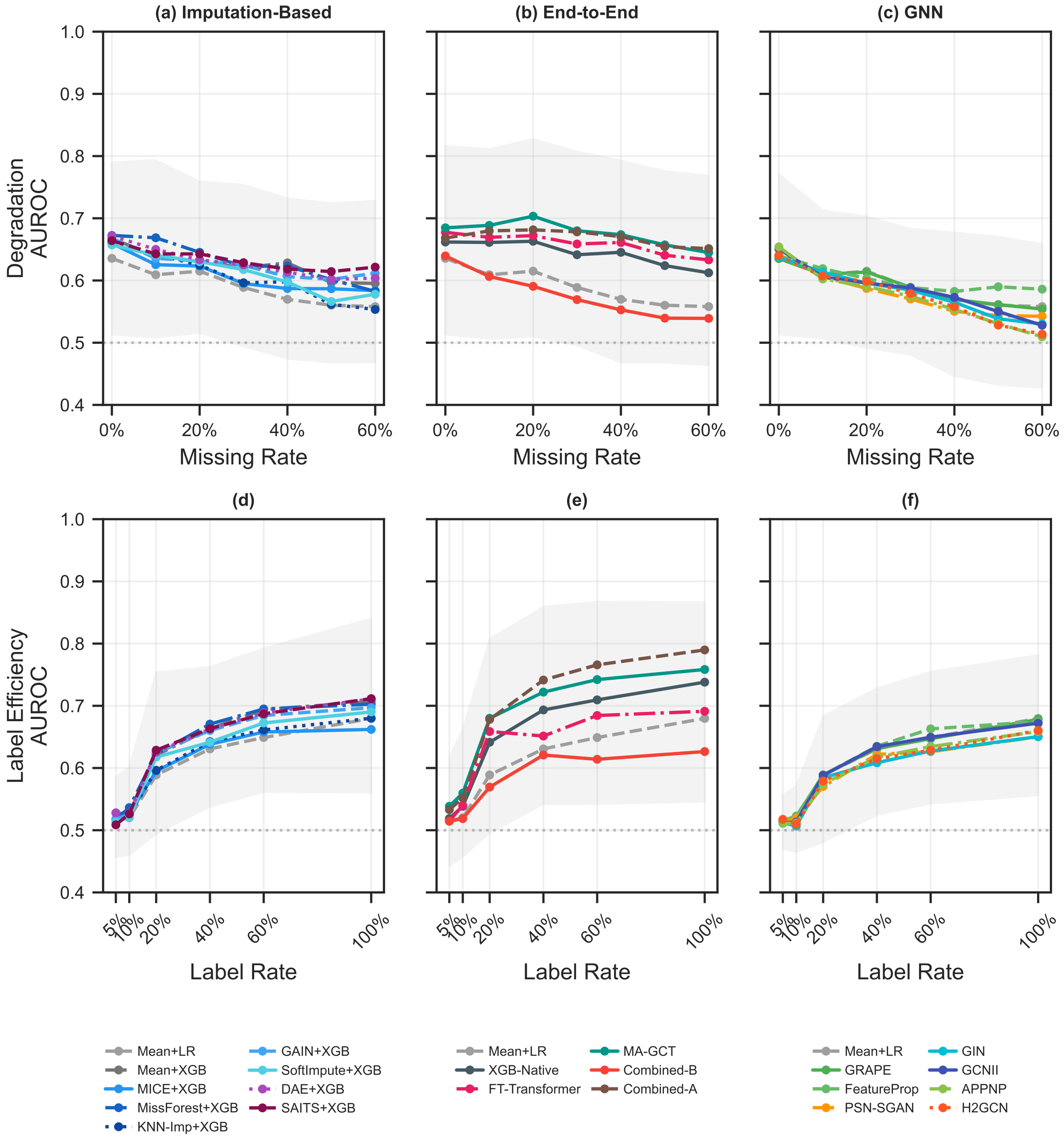

Figure S1: MNAR performance profiles, complementing the MCAR panels in the main text (Figures 5 and 6). (**a–c**) AUROC degradation curves under MNAR at 20% label rate, grouped by method family; the gray envelope shows the overall ±1 s.d. range. (**d–f**) Label efficiency curves under MNAR at 30% missing rate. Mean+LR (dashed gray) appears in all panels as a baseline reference.

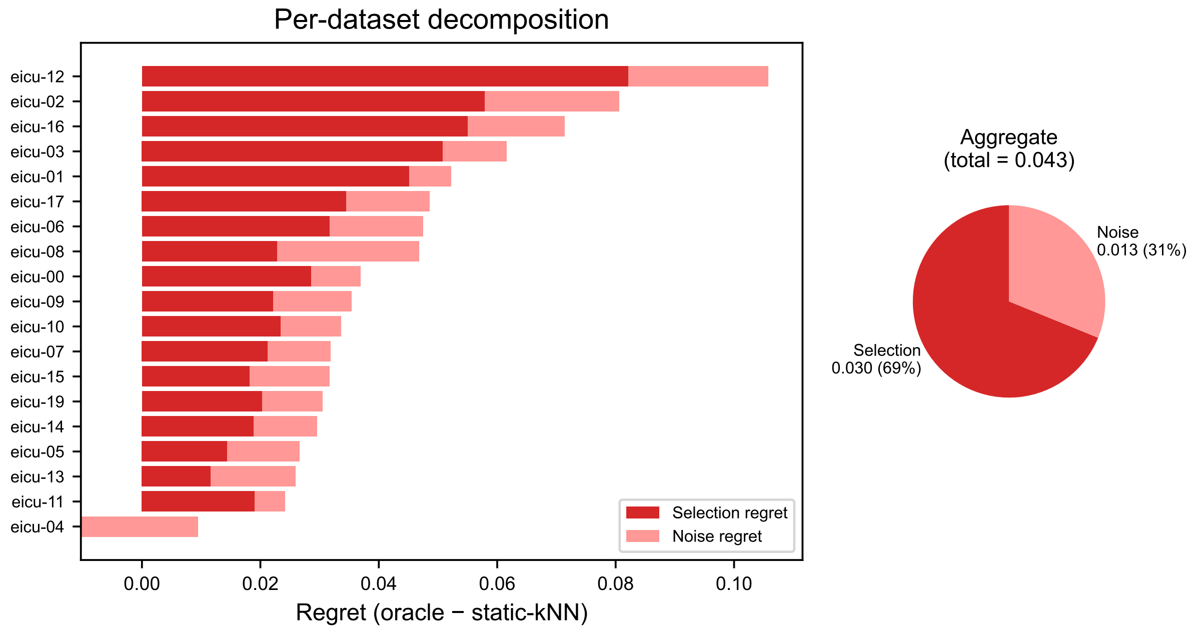

Figure S2: Per-dataset oracle gap decomposition on the augmented store ($N = 83$, 19 base groups). Left: stacked bars show selection regret (choosing the wrong method) and noise regret (winner instability among near-optimal methods) for each base dataset. Right: aggregate composition—selection regret accounts for 66% of the total gap (0.043), noise regret for 34%. Companion to the aggregate analysis in Section 4.7 of the main text.

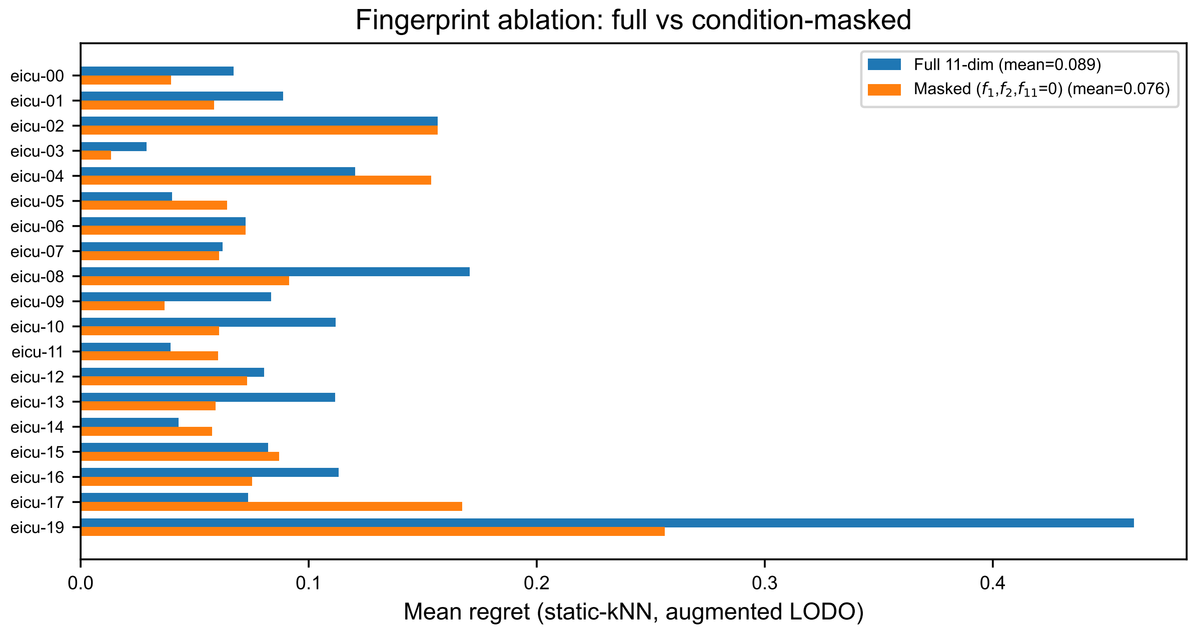

Figure S3: LODO ablation: full fingerprint vs. condition-masked fingerprint (dimensions f_1, f_2, f_{11} set to zero). The ablated static-kNN achieves regret 0.076 vs. 0.089 for the full version, confirming that structural dataset properties (f_3–f_{10}) carry the recommendation signal rather than condition-encoding dimensions.

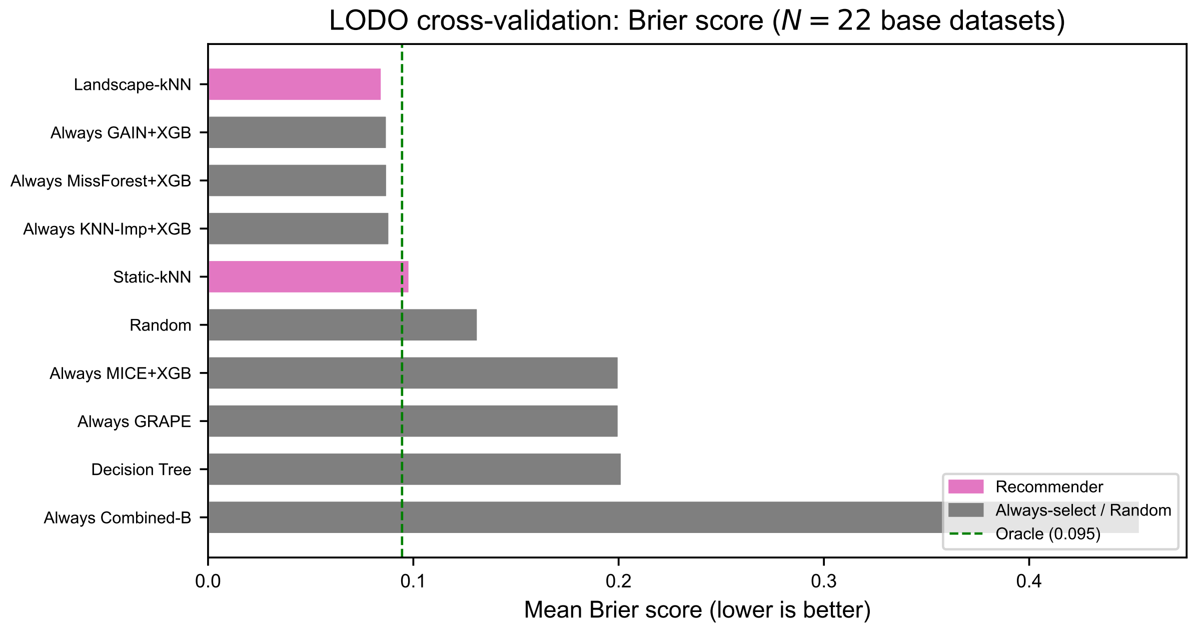

Figure S4: LODO cross-validation evaluated by Brier score ($N = 22$ base datasets, reference condition). Strategies sorted by mean Brier score (lower is better); dashed line marks oracle. Complements the AUROC-based evaluation in Section 4.5 of the main text and tests whether recommender rankings are metric-dependent (Discussion, Section 5.5).

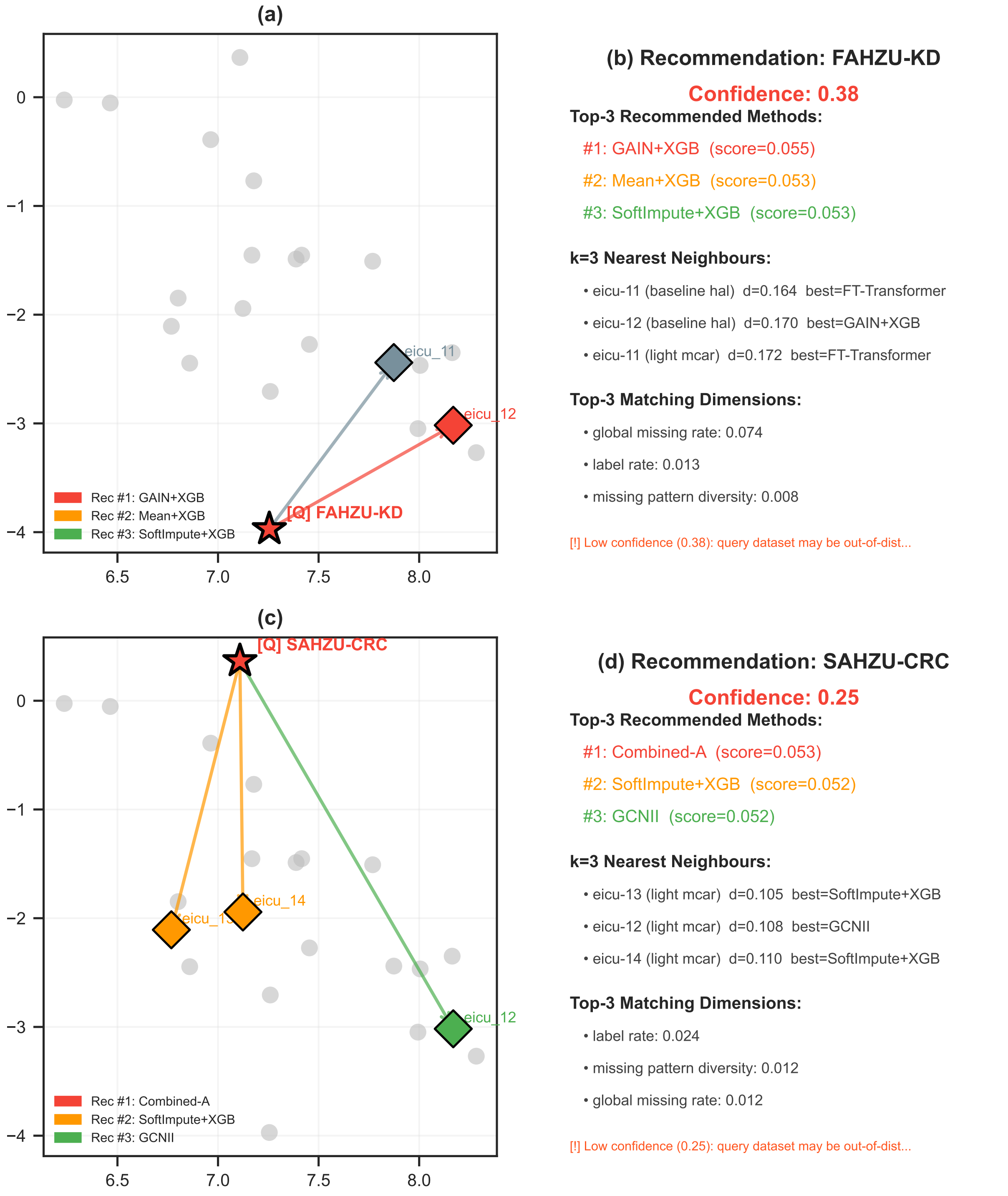

Figure S5: External validation: nearest-neighbour recommendation for FAHZU-Kidney and SAHZU-CRC in fingerprint space. (**a**) t-SNE projection of FAHZU-Kidney (star) and its k=3 nearest neighbours in the augmented knowledge base; neighbour markers are colored by their oracle-best method. (**b**) Recommendation details for FAHZU-Kidney: confidence score, top-3 recommended methods, nearest neighbours with distances and oracle-best labels, and top matching fingerprint dimensions. (**c**, **d**) Same layout for SAHZU-CRC. Distances are computed in the learned 10-dimensional weighted fingerprint space (Section 3.9). Low-confidence warnings indicate the query may be out-of-distribution.

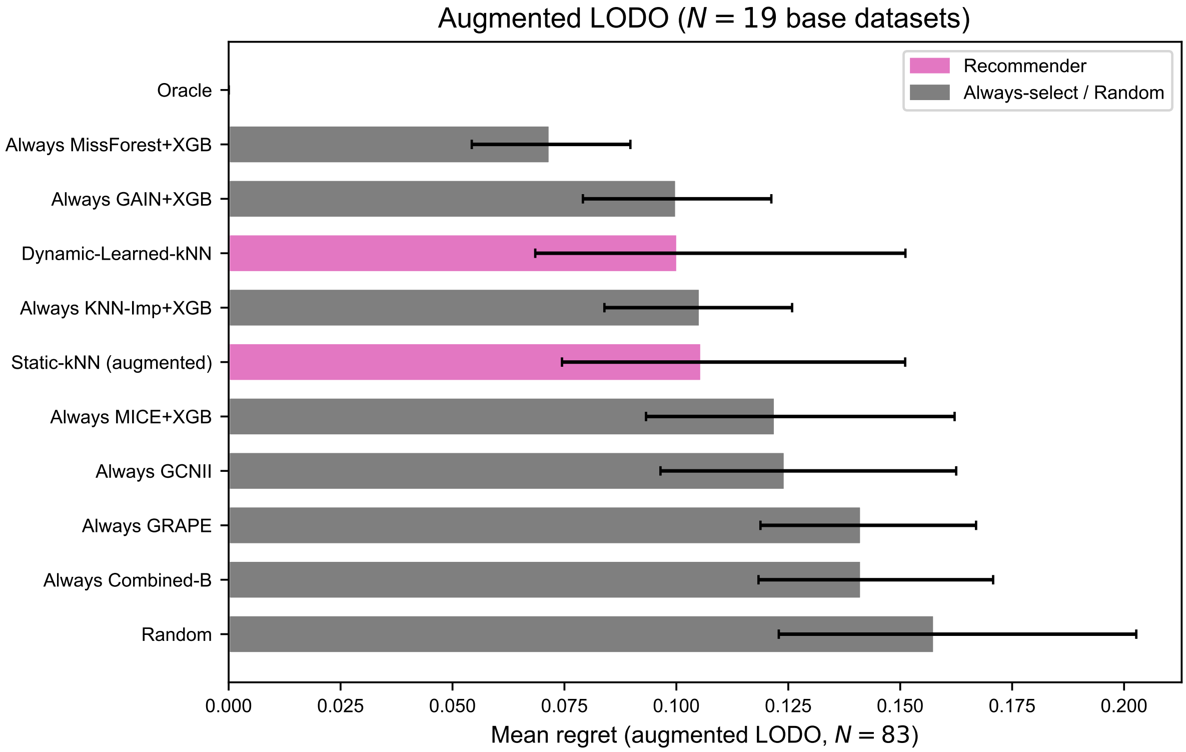

Figure S6: Bootstrap regret comparison across recommender strategies on the augmented LODO ($N = 83$ instances, 19 base groups). Strategies are ranked by mean regret; error bars show block-bootstrap 95% confidence intervals (B = 2,000, base datasets resampled with replacement). Pink bars denote fingerprint-based recommenders; gray bars denote always-select baselines and random selection.

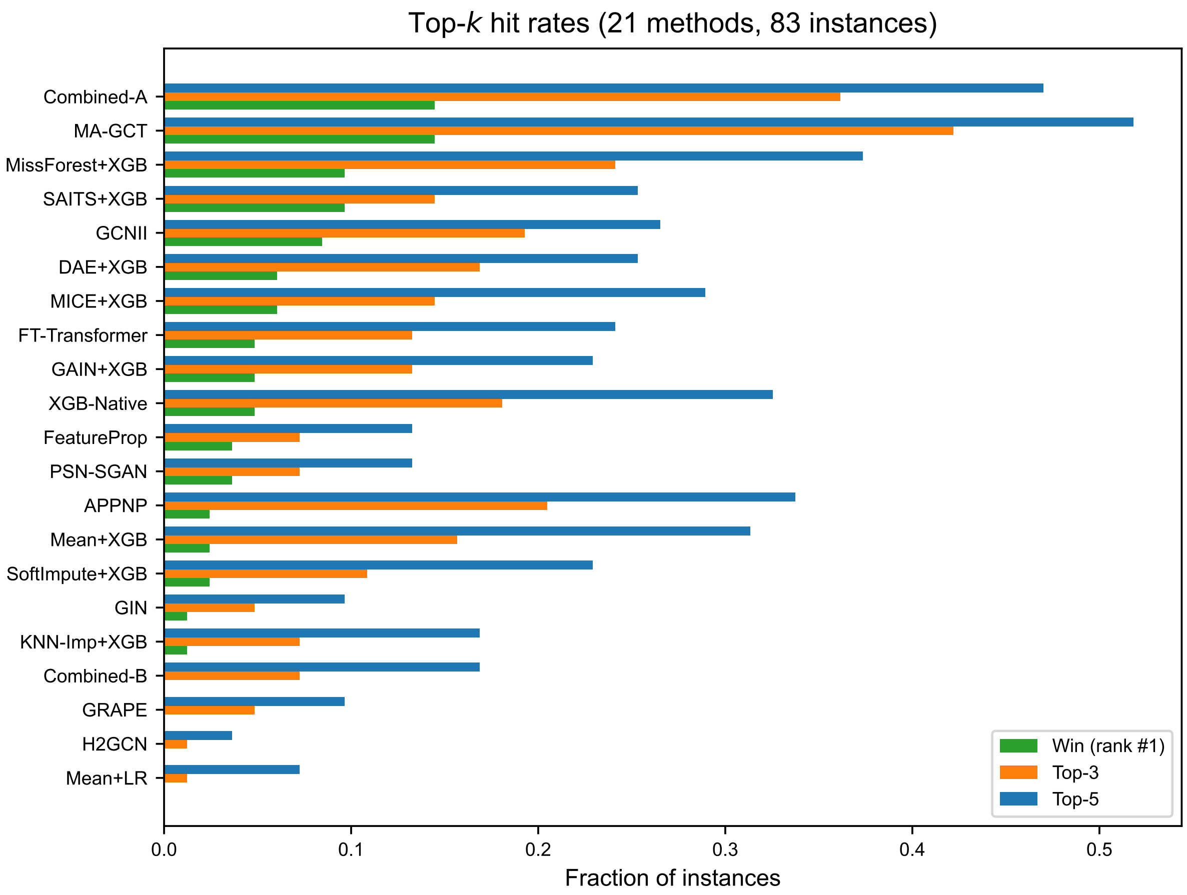

Figure S7: Top-k hit rates across the full condition grid (missing rate × label rate × mechanism). For each of the 21 methods, bars show the win rate (rank #1), top-3 rate, and top-5 rate. Red asterisks mark methods that never win under the reference condition (miss = 0.2, label = 0.4, MCAR).

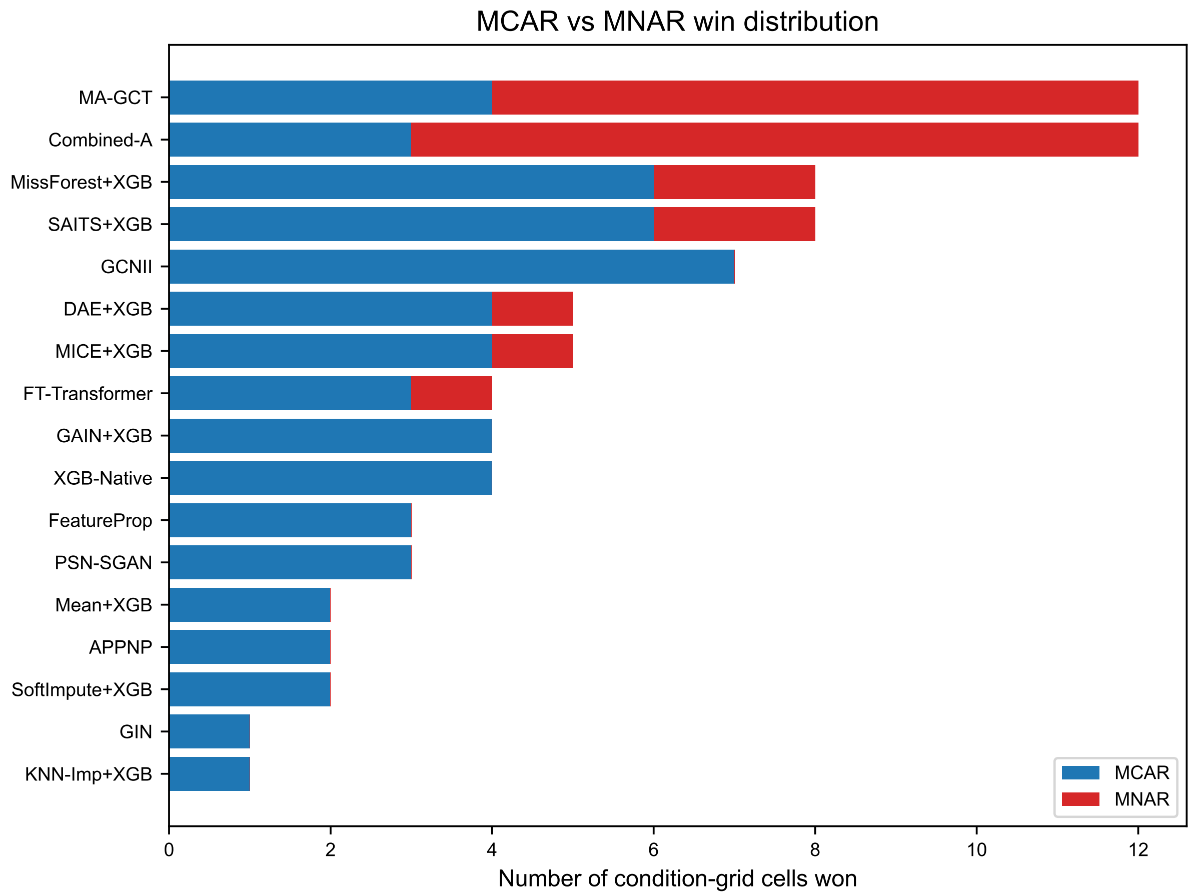

Figure S8: MCAR vs. MNAR win distribution across all 21 methods. Horizontal stacked bars show the number of condition-grid cells each method wins under MCAR (blue) and MNAR (red), sorted by total wins. Missingness-aware methods (MA-GCT, Combined-A) concentrate their wins under MNAR, indicating strong specialization to informative missingness.

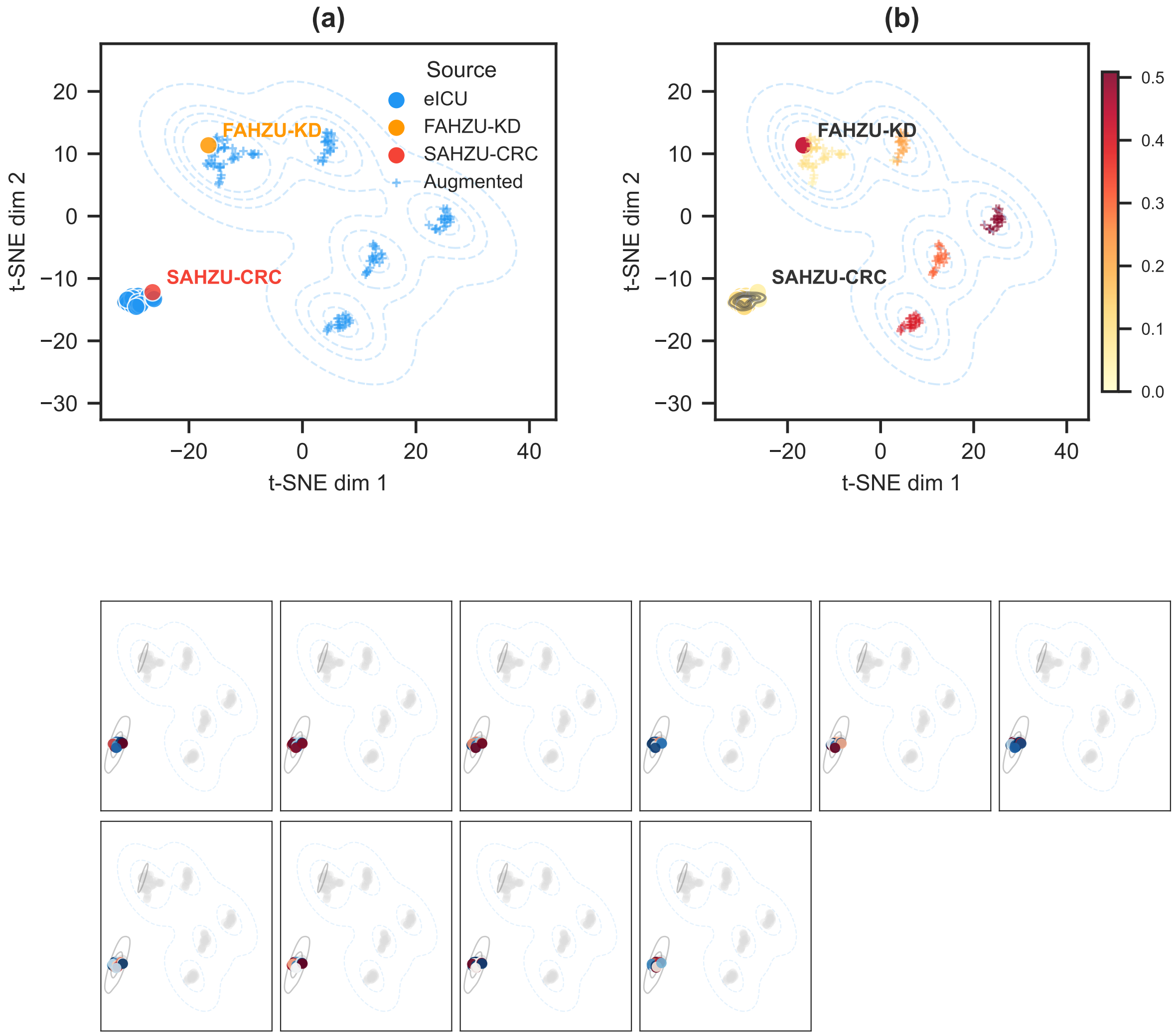

Figure S9: t-SNE visualization of the 11-dimensional fingerprint space. (**a**) Points colored by source: eICU base datasets (blue), augmented instances (light blue crosses), and external cohorts FAHZU-Kidney (orange) and SAHZU-CRC (red). (**b**) Same embedding colored by overall missing rate. Bottom panels show per-dataset neighbourhood detail with confidence ellipses.

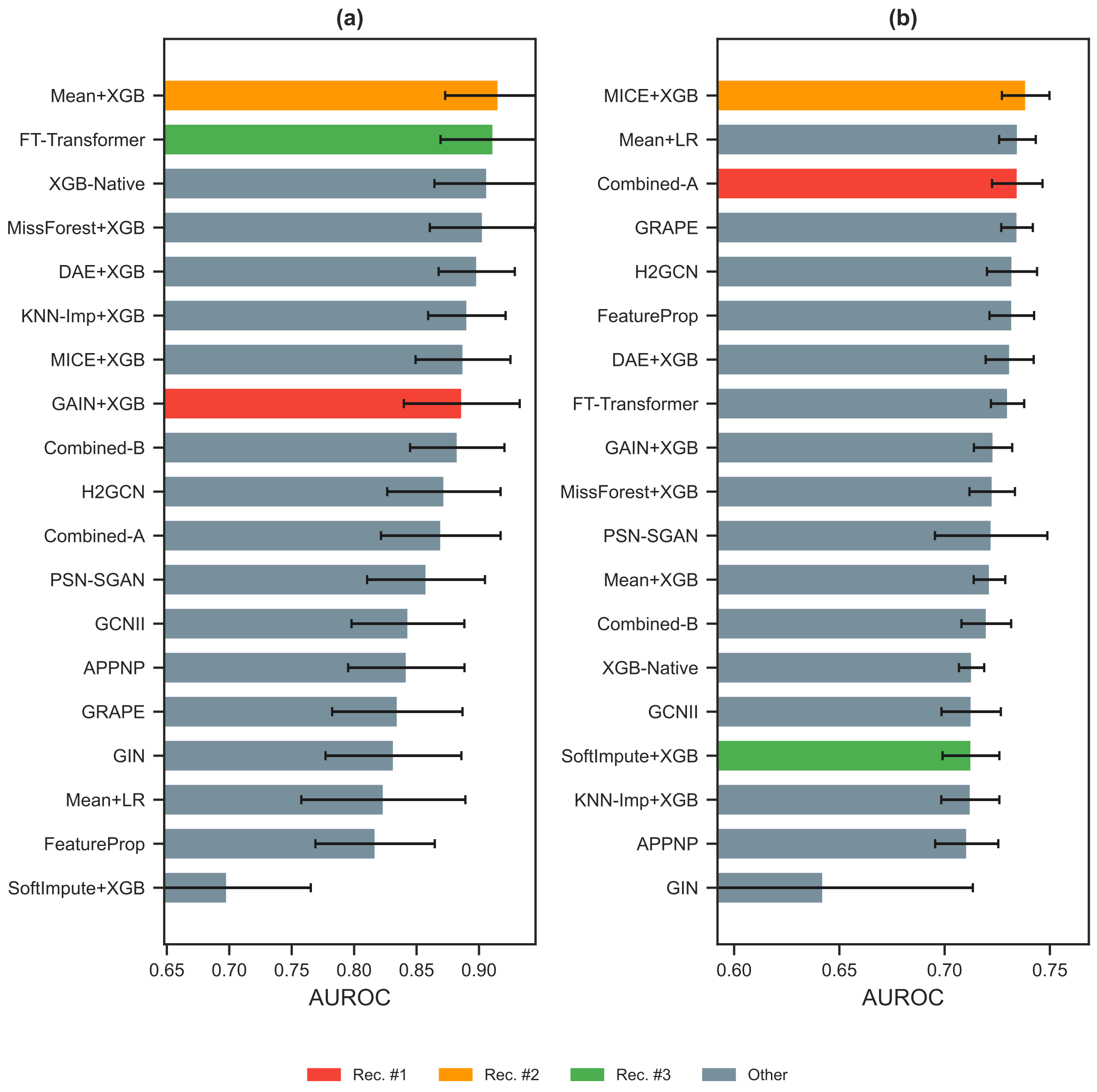

Figure S10: External validation: 19 of 21 methods ranked by AUROC on two independent cohorts (SAITS+XGB and MA-GCT are excluded because they failed to converge on both external cohorts). The recommended method is shown in red. (**a**) FAHZU-Kidney (N = 14,164, 28 features, 44% missing): the recommender selects GAIN+XGB (rank #8). (**b**) SAHZU-CRC (N = 2,080, 18 features, 6% missing): the recommender selects Combined-A (rank #3). Error bars are ±1 s.e. across cross-validation folds.

Section S4. External Validation Details

This section provides the full OOD analysis and per-cohort discussion for the three external validation cohorts (25 subsets; Section 4.8 of the main text).

Table S2: External dataset OOD analysis: FAHZU-Kidney and SAHZU-CRC.

Dimension	eICU range	FAHZU-Kidney	SAHZU-CRC
global_miss (f_1)	0.04–0.13	**0.437** (far OOD)	0.055 (in range)
label_rate (f_2)	1.0	**0.313** (far OOD)	1.0 (in range)
class_imbalance (f_8)	0.02–0.15	∼0.19	**0.488** (far OOD)
max_feature_miss (f_4)	0.37–0.81	**0.968** (far OOD)	0.289 (below range)
Little's MCAR (f_{11})	∼0.0	—	—

Table S3: External dataset OOD analysis: MIMIC-IV (range across 8 ICD-diagnosis groups).

Dimension	eICU range	MIMIC-IV (8 groups)
global_miss (f_1)	0.04–0.13	**0.15–0.29** (OOD)
label_rate (f_2)	1.0	1.0 (in range)
class_imbalance (f_8)	0.02–0.15	0.04–**0.32** (partial OOD)
max_feature_miss (f_4)	0.37–0.81	0.75–**0.95** (in/above range)
Little's MCAR (f_{11})	∼0.0	**0.04–0.24** (MNAR)

SAHZU-CRC ($N = 2{,}080$, 18 features, 5.5% missing, 5-year survival): Dynamic-Learned-kNN recommends the Combined-B pipeline, which achieves **rank #3** among all 21 methods. The dataset is in-distribution on f_1 (global_miss) and f_2 (label_rate) but OOD on f_8 (class_imbalance 0.488 vs. eICU range 0.02–0.15). Despite this shift, the learned weights correctly prioritize missingness-structure features, and the recommendation is near-optimal.

FAHZU-Kidney ($N = 14{,}164$, 28 features, 43.7% missing, 1-year mortality): Dynamic-Learned-kNN recommends GAIN+XGB, achieving rank #8/21. The dataset is far OOD on four fingerprint dimensions simultaneously (Table S2). The rank-#8 result is modest: the extreme distribution shift ($f_1 = 0.437$ vs. eICU max 0.13) places this dataset outside the region where the learned weights, optimized on eICU dynamic distances, can reliably transfer. The oracle method (Mean+XGB) belongs to the same imputation-based family, so the recommendation direction is correct even if the specific method choice is not.

MIMIC-IV (8 ICD-diagnosis groups; $N = 1{,}771$–$24{,}873$; 28 features; 15–29% missing; US multi-center ICU [16]): Using the eICU-only KB with f_1–f_{10}, the recommender selects MICE+XGB for 6 of 8 groups. Per-group results are shown in Table S5. Mean regret is 0.025. The oracle method is Combined-A for 5 groups, Mean+XGB for 2, and XGB-Native for 1. Compared to FAHZU-Kidney and SAHZU-CRC, MIMIC-IV is OOD primarily on global missingness ($f_1 = 0.15$–0.29, all above eICU max 0.13) and on missingness mechanism (f_{11}: strongly MNAR vs. eICU $\approx$ MCAR). Despite this shift, the moderate per-group regrets (5 of 8 groups < 0.02) suggest that the recommender transfers reasonably well when missingness rate is the dominant OOD dimension.

Table S4: Cross-domain KB-source × fingerprint ablation (mean regret across 25 external subsets × 8 degradation conditions). Random-selection baseline: MCAR 0.037, MNAR 0.068, ALL 0.053. Bold = best per KB source. pymfe6 = {`class_conc.sd`, `freq_class.sd`, `class_ent`, `class_conc.mean`, `mad.sd`, `attr_conc.sd`}; pymfe8 = pymfe6 ∪ {`joint_ent.mean`, `min.sd`}. All extracted via pymfe [17] from groups `general`, `statistical`, `info-theory`; selected by KB–cross-domain distribution overlap.

KB source	Fingerprint	Dims	MCAR	MNAR	ALL
eICU-only	f_1–f_{10}	10	0.039	0.092	0.066
eICU-only	**pymfe6**	6	**0.026**	**0.053**	**0.040**
eICU-only	pymfe8	8	**0.026**	0.056	0.041
eICU-only	miss6+pymfe6	12	**0.026**	0.057	0.042
Synthetic	f_1–f_{10}	10	0.032	0.078	0.055
Synthetic	**pymfe6**	6	**0.024**	**0.026**	**0.025**
Synthetic	pymfe8	8	0.027	0.028	0.028
Synthetic	miss6+pymfe6	12	0.033	0.055	0.044
eICU+Synth	f_1–f_{10}	10	0.040	0.087	0.064
eICU+Synth	**pymfe6**	6	0.029	**0.036**	**0.032**
eICU+Synth	pymfe8	8	0.029	0.039	0.034
eICU+Synth	miss6+pymfe6	12	**0.026**	0.057	0.041

Table S5: MIMIC-IV per-group recommender results (eICU-only KB, f_1–f_{10} fingerprint).

Group	N	Miss%	Oracle	Oracle AUC	Recommended	Regret
Circulatory	24,873	24.4	Combined-A	0.860	MICE+XGB	0.040
Digestive	5,793	19.3	Combined-A	0.852	MICE+XGB	0.010
Endocrine	1,798	25.4	Mean+XGB	0.871	APPNP	0.073
Infectious	6,673	15.2	Combined-A	0.820	MICE+XGB	0.003
Injury	9,747	27.2	Mean+XGB	0.829	MICE+XGB	0.016
Neoplasm	5,475	26.5	XGB-Native	0.851	MICE+XGB	0.012
Nervous	1,771	29.2	Combined-A	0.851	MICE+XGB	0.018
Respiratory	3,967	23.8	Combined-A	0.782	MICE+XGB	0.024
**Mean**				0.840		0.025

[1] Li, R.; Tian, Y.; Shen, Z.; Li, J.; Li, J.; Ding, K.; Li, J. Improving an Electronic Health Record-Based Clinical Prediction Model Under Label Deficiency: Network-Based Generative Adversarial Semisupervised Approach. *JMIR Med. Inform.* **2023**, *11*, e47862. https://doi.org/10.2196/47862.

[2] Choi, E.; Xu, Z.; Li, Y.; Dusenberry, M.W.; Flores, G.; Xue, E.; Dai, A.M. Learning the graphical structure of electronic health records with graph convolutional transformer. In Proceedings of the AAAI Conference on Artificial Intelligence, New York, NY, USA, 7–12 February 2020; Volume 34, pp. 606–613. https://doi.org/10.1609/aaai.v34i01.5400.

[3] Buuren, S.V.; Groothuis-Oudshoorn, K. mice: Multivariate Imputation by Chained Equations in R. *J. Stat. Softw.* **2011**, *45*, 1–67. https://doi.org/10.18637/jss.v045.i03.

[4] Stekhoven, D.J.; Bühlmann, P. MissForest—Non-parametric missing value imputation for mixed-type data. *Bioinformatics* **2012**, *28*, 112–118. https://doi.org/10.1093/bioinformatics/btr597.

[5] Yoon, J.; Jordon, J.; van der Schaar, M. GAIN: Missing data imputation using generative adversarial nets. In *Proceedings of the 35th International Conference on Machine Learning*; Dy, J., Krause, A., Eds.; PMLR; 2018; Volume 80, pp. 5689–5698.

[6] Mazumder, R.; Hastie, T.; Tibshirani, R. Spectral Regularization Algorithms for Learning Large Incomplete Matrices. *J. Mach. Learn. Res.* **2010**, *11*, 2287–2322.

[7] Gondara, L.; Wang, K. MIDA: Multiple Imputation Using Denoising Autoencoders. In *Proceedings of the Advances in Knowledge Discovery and Data Mining*; Lecture Notes in Computer Science; Springer International Publishing: Cham, Switzerland, 2018; Volume 10939, pp. 260–272. https://doi.org/10.1007/978-3-319-93040-4_21.

[8] You, J.; Ma, X.; Ding, D.Y.; Kochenderfer, M.J.; Leskovec, J. Handling missing data with graph representation learning. *Adv. Neural Inf. Process. Syst.* **2020**, *33*, 19075–19087.

[9] Rossi, E.; Kenlay, H.; Gorinova, M.I.; Chamberlain, B.P.; Dong, X.; Bronstein, M.M. On the Unreasonable Effectiveness of Feature Propagation in Learning on Graphs with Missing Node Features. In *Learning on Graphs Conference*; 2022.

[10] Xu, K.; Hu, W.; Leskovec, J.; Jegelka, S. How powerful are graph neural networks? In Proceedings of the International Conference on Learning Representations, New Orleans, LA, USA, 6–9 May 2019.

[11] Chen, M.; Wei, Z.; Huang, Z.; Ding, B.; Li, Y. Simple and deep graph convolutional networks. In Proceedings of the 37th International Conference on Machine Learning (ICML), Virtual, 13–18 July 2020; Volume 119, pp. 1725–1735.

[12] Gasteiger, J.; Bojchevski, A.; Günnemann, S. Predict then propagate: Graph neural networks meet personalized PageRank. In Proceedings of the International Conference on Learning Representations (ICLR), New Orleans, LA, USA, 6–9 May 2019.

[13] Zhu, J.; Yan, Y.; Zhao, L.; Heimann, M.; Akoglu, L.; Koutra, D. Beyond homophily in graph neural networks: Current limitations and effective designs. *Adv. Neural Inf. Process. Syst.* **2020**, *33*, 7793–7804.

[14] Chen, T.; Guestrin, C. XGBoost: A Scalable Tree Boosting System. In Proceedings of the 22nd ACM SIGKDD International Conference on Knowledge Discovery and Data Mining, San Francisco, CA, USA, 13–17 August 2016; pp. 785–794. https://doi.org/10.1145/2939672.2939785.

[15] Gorishniy, Y.; Rubachev, I.; Khrulkov, V.; Babenko, A. Revisiting Deep Learning Models for Tabular Data. *arXiv* **2021**. https://doi.org/10.48550/ARXIV.2106.11959.

[16] Johnson, A.E.W.; Bulgarelli, L.; Shen, L.; Gayles, A.; Shammout, A.; Horng, S.; Pollard, T.J.; Hao, S.; Moody, B.; Gow, B.; et al. MIMIC-IV, a freely accessible electronic health record dataset. *Sci. Data* **2023**, *10*, 1. https://doi.org/10.1038/s41597-022-01899-x.

[17] Rivolli, A.; Garcia, L.P.F.; Soares, C.; Vanschoren, J.; de Carvalho, A.C.P.L.F. Meta-features for meta-learning. *Knowl.-Based Syst.* **2022**, *240*, 108101. https://doi.org/10.1016/j.knosys.2021.108101.

[18] Du, W.; Côté, D.; Liu, Y. SAITS: Self-attention-based imputation for time series. *Expert Syst. Appl.* **2023**, *219*, 119619. https://doi.org/10.1016/j.eswa.2023.119619.
